# Atorvastatin Mitigates Hypercholesterolemia-Induced Alterations in Cerebral Mitochondrial Dehydrogenases in Rats

**DOI:** 10.3390/ijms27156791

**Published:** 2026-07-29

**Authors:** Malgorzata Belczyk, Anna Gawedzka, Jagoda Drag, Oliwia Stelmach, Malgorzata Knapik-Czajka

**Affiliations:** Department of Biochemical Analytics, Faculty of Pharmacy, Jagiellonian University Medical College, 30-688 Krakow, Poland; malgorzata.belczyk@uj.edu.pl (M.B.); anna.gawedzka@uj.edu.pl (A.G.); jagoda.drag@uj.edu.pl (J.D.); oliwia.1.stelmach@student.uj.edu.pl (O.S.)

**Keywords:** α-ketoglutarate dehydrogenase (α-KGDH), isocitrate dehydrogenase (IDH), glutamate dehydrogenase (GDH), hypercholesterolemia, rats, brain, glutamate

## Abstract

Statins are widely used lipid-lowering agents for the prevention and treatment of cardiovascular disease. Atorvastatin, one of the most frequently used statins, was reported to influence mitochondrial function, including the regulation of mitochondrial enzyme activity. Our recent study demonstrated that short-term treatment with atorvastatin effectively counteracted the alterations in α-ketoglutarate dehydrogenase (α-KGDH) induced by a high-cholesterol diet. Since α-KGDH and isocitrate dehydrogenase (IDH) together with glutamate dehydrogenase (GDH) are functionally interconnected, we hypothesized that atorvastatin modulates their activity in the brain. The study aimed to evaluate the effect of short-term atorvastatin treatment on cerebral mitochondrial dehydrogenases α-KGDH, IDH and GDH in hypercholesterolemic rats. Atorvastatin at dose 20 mg/kg b·wt/day (HC+A, *n* = 8) or vehicle (HC, *n* = 8) were administered to HC rats for 3 weeks. The control group was fed a standard diet (ST, *n* = 8). α-KGDH activity and relative protein abundance of its subunits were evaluated. Similarly, IDH and GDH activities as well as their relative protein levels were determined. In addition, the level of cerebral glutamate was measured. The study showed that hypercholesterolemia induced elevation of α-KGDH and IDH activities and reduction in GDH activity and glutamate level. Treatment with atorvastatin mitigated these metabolic disturbances, highlighting its potentially favorable effect in the brain.

## 1. Introduction

Statins, inhibitors of HMG-CoA reductase, are widely prescribed lipid-lowering drugs, applied in the prevention, as well as the treatment of the cardiovascular disease (CVD) [1]. Mitochondria have emerged as key cellular targets of statin action. Atorvastatin, one of the most extensively prescribed statins worldwide, has been shown to modulate mitochondrial function including altering coenzyme Q10 pool and cellular redox balance [2,3]. In addition, previous studies demonstrated that atorvastatin influences several mitochondrial enzymes, comprising those of the 2-oxoacid dehydrogenase family [4,5,6].

One of the members of the mitochondrial 2-oxoacid dehydrogenase family is α-ketoglutarate dehydrogenase complex (α-KGDH, also known as 2-OGDH, 2-oxoglutarate dehydrogenase) [7]. The α-KGDH complex is composed of multiple copies of three catalytic subunits: α-ketoglutarate dehydrogenase (OGDH, E1), dihydrolipoamide succinyl transferase (DLST, E2), and dihydrolipoamide dehydrogenase (DLD, E3). The E1 subunit catalyzes the irreversible decarboxylation of α-ketoglutarate (α-KG, also known as 2-oxoglutarate) and defines both the initial and rate-limiting step of the complex. The E2 subunit catalyzes the transfer of the succinyl group to coenzyme A, forming succinyl-CoA, while the E3 subunit regenerates the oxidized lipoamide with concomitant reduction of NAD+ to NADH. The E2 subunits form the structural core of the complex, to which E1 and E3 subunits are loosely attached. In mammalian cells, substrate specificity is determined by the E1 and E2 components, whereas the E3 subunit is shared among different 2-oxoacid dehydrogenase complexes [8,9].

As a key enzyme of the tricarboxylic acid (TCA) cycle (also known as Krebs cycle), α-KGDH plays an essential role in cerebral energy metabolism. The complex is functionally associated with isocitrate dehydrogenase (IDH), another regulatory enzyme of the TCA cycle, which catalyzes the conversion of isocitrate to α-KG, a substrate for α-KGDH.

In addition, by regulating α-KG availability, both α-KGDH (an α-KG-consuming enzyme) and IDH (an α-KG-producing enzyme) modulate glutamate dehydrogenase (GDH) activity and consequently affect glutamate homeostasis. GDH catalyzes the reversible conversion of α-KG to glutamate. In the brain, the reductive amination of α-KG to glutamate contributes to cerebral glutamate homeostasis by replenishing glutamate, the principal excitatory neurotransmitter.

Our recent study demonstrated that hypercholesterolemia reduced α-KGDH and GDH (in the direction of oxidative deamination of glutamate to α-KG) functions in rat’s liver. Short-term (3 weeks) treatment with atorvastatin (20 mg/kg/b·wt/day) effectively counteracted the alterations in liver α-KGDH and GDH induced by a high-cholesterol diet, thereby highlighting beneficial action of atorvastatin beyond its lipid-lowering effect [10]. The influence of atorvastatin on cerebral α-KGDH and GDH activities in hypercholesterolemic rats has not been studied so far. Given that atorvastatin has been shown to exert a favorable effect on hepatic dehydrogenases, we hypothesized that atorvastatin affects cerebral α-KGDH in rats with hypercholesterolemia.

The main aim of the present study was to determine the effect of short-term (3 weeks) treatment with atorvastatin (20 mg/kg b·wt/day) on cerebral α-KGDH in hypercholesterolemic rats. Another purpose was to investigate the atorvastatin effect on enzymes that are functionally connected with α-KGDH complex such as IDH and GDH. Therefore, we determined α-KGDH activity and assessed the relative protein abundance of the E1 and E2 subunits of the complex. The E3 subunit was not evaluated as it is a shared component of enzymes belonging to 2-oxoacid dehydrogenase family. In addition, the activities of GDH and IDH, as well as their relative protein levels, were measured. Moreover, the level of glutamate in the whole brain tissue was measured.

## 2. Results

### 2.1. Body Weight, Food Consumption and Serum Cholesterol Level

As we previously reported [11], no significant differences were found in the final body weight among all experimental groups. HC rats exhibited significantly greater food consumption and energy intake than those in the ST group (*p* < 0.05). In addition, no significant differences were observed in the weights of subcutaneous adipose tissue (6.7 ± 1.1 g; 7.6 ± 1.4 g; 6.3 ± 1.0 g for ST; HC and HC+A group, respectively; *p* > 0.05). The HC group showed significantly higher serum LDL-C than the ST group (*p* < 0.01), while atorvastatin administration significantly reduced both TC and LDL-C levels compared with the HC group, confirming lipid-lowering effect of the drug (*p* < 0.05 and *p* < 0.01, respectively). These results are reported with details and discussed in our recently published papers [10,11].

### 2.2. The Effect of Atorvastatin on Cerebral α-KGDH Activity

**Figure 1:**
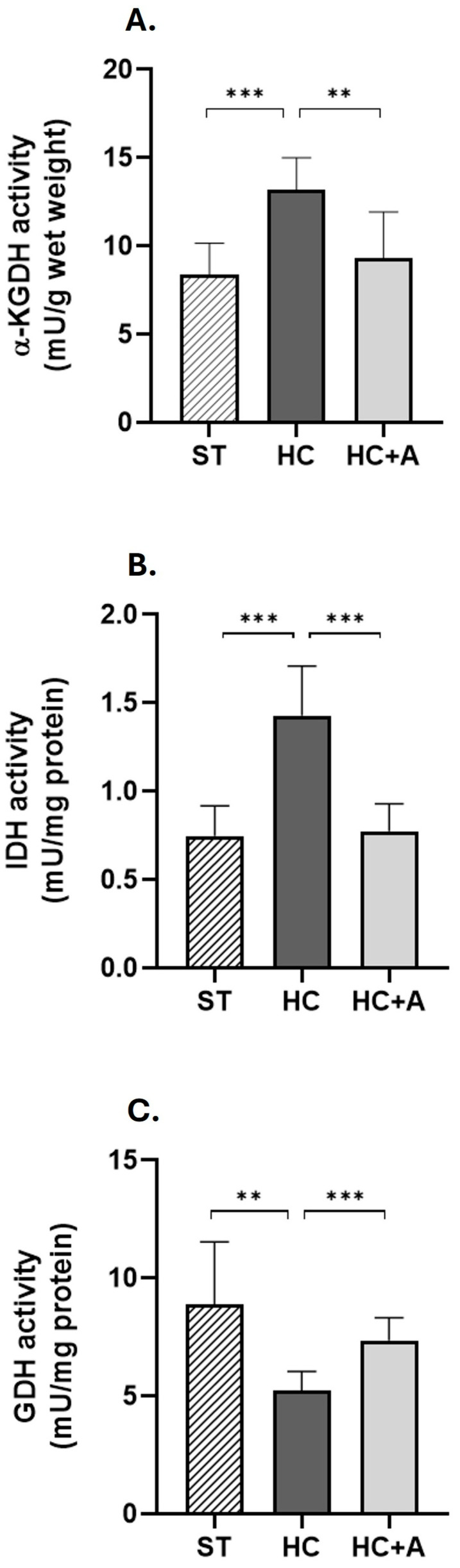
The effect of atorvastatin on cerebral α-KGDH (**A**), IDH (**B**) and GDH (**C**) activities. Study groups were rats fed standard diet (ST, *n* = 8), rats fed cholesterol-enriched diet (HC, *n* = 8), rats fed HC diet treated with atorvastatin (HC+A, *n* = 8). Data are presented as means ± SD, (*** *p* < 0.001, ** *p* < 0.01).

Cerebral α-KGDH activity increased in response to feeding rats with a cholesterol-enriched diet as compared to rats fed a standard diet (by 57%, *p* < 0.001). α-KGDH activity in HC+A rats was lower than in HC animals (by 30%, *p* < 0.01) (Figure 1A).

### 2.3. The Effect of Atorvastatin on Cerebral IDH Activity

Cerebral IDH activity increased in hypercholesterolemic rats as compared to rats fed standard diet (by 91%, *p* < 0.001). In rats administered with atorvastatin, IDH activity was significantly lower than in HC animals (by 46%, *p* < 0.01) (Figure 1B).

### 2.4. The Effect of Atorvastatin on Cerebral GDH Activity

Cerebral GDH activity decreased in response to feeding rats with a cholesterol-enriched diet as compared to rats fed a standard diet (by 41%, *p* < 0.01). In atorvastatin-treated rats, GDH activity was higher than in HC animals (by 37%, *p* < 0.001) (Figure 1C).

### 2.5. The Effect of Atorvastatin on Cerebral E1 and E2 Protein Levels

**Figure 2:**
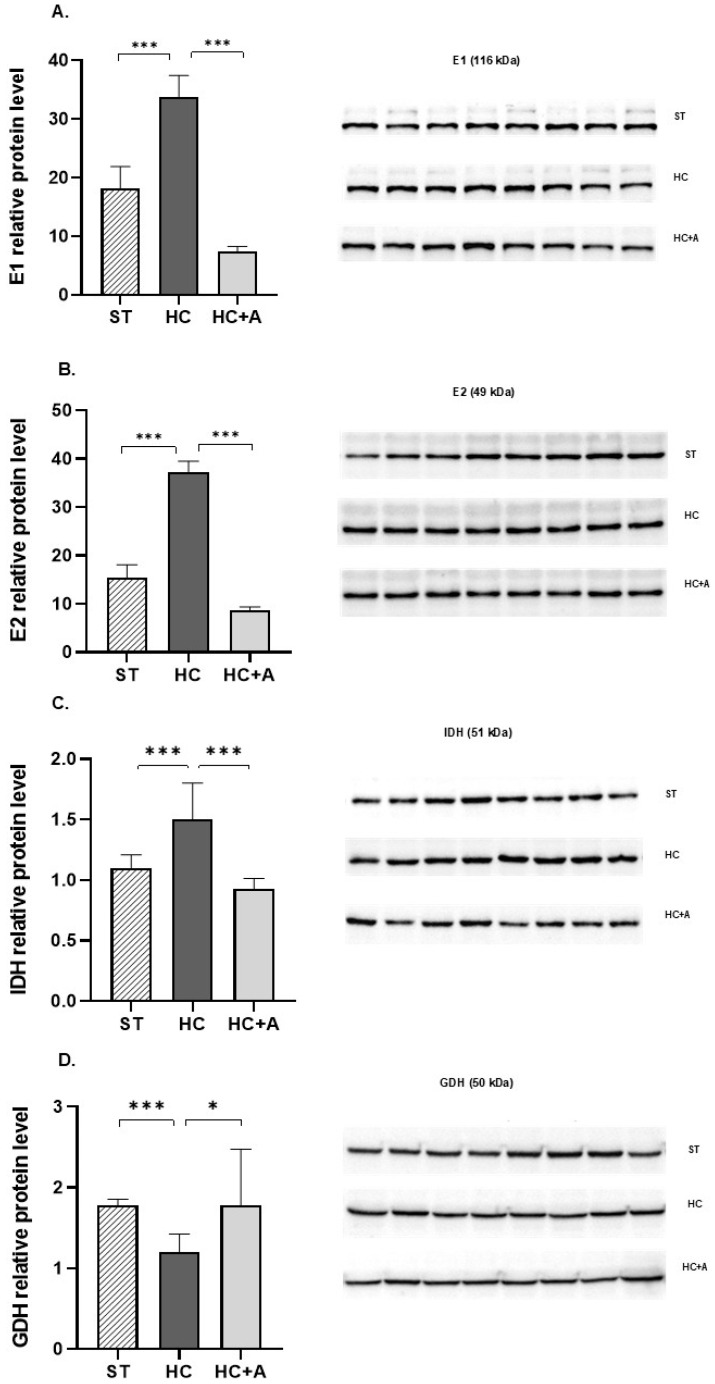
The effect of atorvastatin on cerebral E1 (**A**) and E2 (**B**) α-KGDH subunits, IDH (**C**) and GDH (**D**) protein level. Study groups were rats fed standard diet (ST, *n* = 8), rats fed cholesterol-enriched diet (HC, *n* = 8) and rats fed HC diet treated with atorvastatin (HC+A, *n* = 8), (* *p* < 0.05 *** *p* < 0.001). Left panel: bar graph displaying means ± SD (**A**) and medians with interquartile ranges (**B**–**D**). Right panel: representative immunoblot.

As shown in Figure 2A, HC rats exhibited a significant elevation in cerebral E1 protein in comparison to those in the ST group (by 86%, *p* < 0.001). Atorvastatin administration to rats fed a cholesterol-enriched diet led to downregulation of E1 expression (by 78% *p* < 0.001).

As shown in Figure 2B, HC rats displayed an increase in cerebral E2 protein level compared to the ST group (by 119%, *p* < 0.001). Administration of atorvastatin to hypercholesterolemic rats resulted in a significant reduction in E2 expression (by 76%, *p* < 0.001).

### 2.6. The Effect of Atorvastatin on Cerebral IDH Protein Level

As shown in Figure 2C, HC rats presented a significant increase in cerebral GDH protein in comparison to those in the ST group (by 37%, *p* < 0.001). Rats fed a cholesterol-enriched diet and treated with atorvastatin demonstrated lower GDH protein expression levels (by 40%, *p* < 0.001).

### 2.7. The Effect of Atorvastatin on Cerebral GDH Protein Level

As shown in Figure 2D, HC rats exhibited a significant decline in cerebral GDH protein in comparison to those in the ST group (by 31%, *p* < 0.001). Atorvastatin treatment resulted in upregulation of GDH protein expression (by 13%, *p* < 0.001).

### 2.8. The Effect of Atorvastatin on Cerebral Glutamate Level

**Figure 3:**
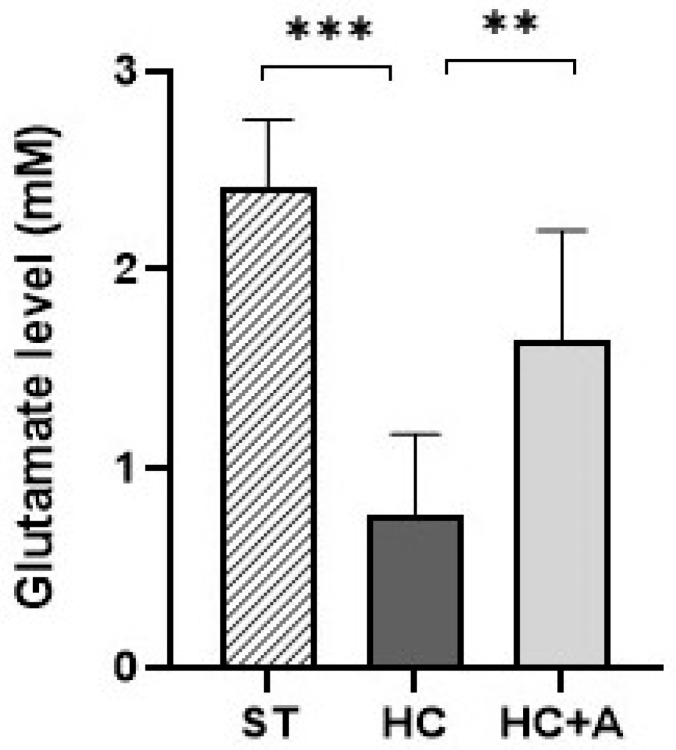
The effect of atorvastatin on cerebral glutamate level. Study groups were rats fed standard diet (ST, *n* = 8), rats fed cholesterol-enriched diet (HC, *n* = 8) and rats fed HC diet treated with atorvastatin (HC+A, *n* = 8). Data are presented as means ± SD, (** *p* < 0.01, *** *p* < 0.001).

Glutamate levels measured in the whole the brain tissue homogenates declined in response to feeding rats with a cholesterol-enriched diet as compared to rats fed a standard diet (by 68%, *p* < 0.001). Glutamate concentration in HC+A rats was significantly higher than in the HC group (by 113%, *p* < 0.01) (Figure 3).

### 2.9. Correlation Between Glutamate Level and Dehydrogenases Activities

Correlation analysis revealed a significant association between glutamate level and α-KGDH activity; a significant negative correlation was identified in ST group (r = −0.87, *p* < 0.05). No significant correlations between glutamate levels and GDH or IDH activity were observed in any of the experimental groups (*p* > 0.05).

## 3. Discussion

To the best of the authors’ knowledge, this is the first report evaluating the effect of atorvastatin on cerebral mitochondrial enzymes, including α-KGDH, IDH and GDH. The present study provided evidence that hypercholesterolemia increased the cerebral activity of α-KGDH. Stimulation of α-KGDH activity in HC rats corresponded to the increase in protein expression of the E1 and E2 subunits of the complex.

Increased α-KGDH activity was shown to be one of the adaptive mechanisms that alleviate general perturbations of mitochondrial homeostasis occurring in response to the metabolic stress [12]. In addition, data provided by Graf et al. [13] demonstrated the stress-dependent activation of the initial steps of the TCA cycle, including α-KGDH in the brain. The authors concluded that the increased flux through α-KGDH is necessary for an effective stress response. Feeding animals a high-cholesterol diet was shown to induce metabolic disturbances, including impaired mitochondrial function [14]. Therefore, stimulation of α-KGDH complex in HC rats may represent a compensatory response to metabolic stress aimed at maintaining proper mitochondrial function and mitigating disturbances induced by a cholesterol-enriched diet.

**Figure 4:**
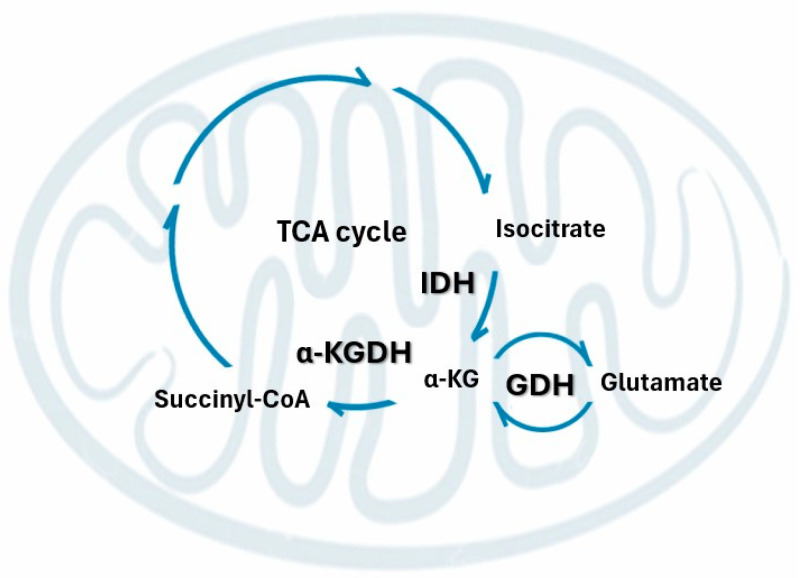
Schematic overview of functional interconnection among α-KGDH, IDH and GDH.

α-KGDH is functionally linked to IDH (Figure 4), another enzyme that may act as a first-line, rapid-response mitochondrial component supporting cellular survival under metabolic stress [15]. Therefore, we hypothesized that alterations in α-KGDH activity may be associated with changes in cerebral IDH activity. As expected, the upregulation of α-KGDH in HC rats was accompanied by an increase in IDH activity and its protein expression.

α-KGDH and IDH by determining the level of α-KG are also involved in glutamate metabolism (Figure 4), a TCA cycle-related metabolite and one of the major neurotransmitters in the brain [16,17,18]. Within the brain, reductive amination of α-KG catalyzed by GDH is one of the sources of glutamate [19]. Although this pathway is not considered a primary source of cerebral glutamate, it contributes to the regulation of glutamate level under specific metabolic conditions [20].

In the HC group, a reduction in the cerebral glutamate level measured in the whole brain tissue homogenates was observed. This change may be linked to attenuated GDH function, as both the activity and protein expression were downregulated in hypercholesterolemic rats. Such a decrease in GDH activity may result from alternations in the levels of allosteric inhibitors of the enzyme, such as GTP and ATP [21]. GTP or ATP levels are dependent on TCA cycle activity [22]. Since α-KGDH and IDH catalyze key regulatory steps of the TCA cycle, the increased activity of these enzymes in HC rats likely indicated higher ATP and GTP levels [23]. Therefore, it is plausible that the increase in α-KGDH and IDH activity in HC rats led to the downregulation of GDH. Additionally, because both α-KGDH and GDH utilize α-KG as a substrate, increased α-KGDH activity may further limit α-KG availability for GDH, reinforcing the reduction in its activity.

A significant negative correlation between glutamate level and α-KGDH activity was identified in the ST group, indicating that these parameters are inversely related under physiological conditions. No correlation was found in HC and HC+A groups. The results suggest that hypercholesterolemia disrupts the physiological correlation and the atorvastatin treatment did not restore this association.

Glutamate is the principal excitatory neurotransmitter in the mammalian central nervous system and mediates most of the fast synaptic transmission [24]. In addition, it serves as the precursor for another neurotransmitter, gamma-aminobutyric acid (GABA). Therefore, reduced glutamate availability in HC rats could impair excitatory/inhibitory signaling, potentially affecting synaptic transmission and brain function.

The present study demonstrated that atorvastatin had a beneficial effect in hypercholesterolemic rats. The treatment of HC rats with atorvastatin led to a decline in cerebral α-KGDH activity, which corresponded to the reduced E1 and E2 subunit protein expression of the complex. This change coincided with reduced IDH activity and its protein level. These findings indicate coordinated regulation of α-KGDH and IDH in response to the atorvastatin treatment. Moreover, the increase in GDH activity and its protein level were observed in atorvastatin treated rats. It is tempting to speculate that reduced α-KGDH activity and thus diminished α-KG utilization by the complex could facilitate GDH-dependent glutamate synthesis by increasing the substrate availability for this enzyme. GDH activation was reported to promote neuroprotective effects [25]. Therefore, the increase in GDH activity induced by atorvastatin may represent an additional beneficial mechanism for counteracting hypercholesterolemia-induced disturbances. Furthermore, the present study demonstrated that atorvastatin treatment normalized the cerebral glutamate level in brain tissue. Since maintaining glutamate homeostasis is fundamental for proper brain function [26], it is reasonable to assume that these changes could also contribute to the improvement in brain metabolism.

To our knowledge, studies evaluating the effects of atorvastatin on selected dehydrogenases in different tissues of hypercholesterolemic rats are scarce, and the available evidence is limited to the liver. A recent study demonstrated that hypercholesterolemia decreased α-KGDH activity in rat hepatic tissue [10]. In contrast, the present study showed increased α-KGDH activity in the brain. Considering the above, these results point to the tissue-specific regulation of the α-KGDH complex in response to hypercholesterolemia.

In the liver, hypercholesterolemia lowered GDH activity similarly to brain tissue. However, GDH activity in the liver was assessed in the oxidative deamination direction using glutamate as the substrate, whereas in the brain it was measured in the reductive amination direction using α-KG as the substrate. The methodological difference reflects the distinct physiological role of GDH in these tissues. Consequently, the GDH results obtained in the two tissues cannot be directly compared.

Atorvastatin effectively counteracted hypercholesterolemia-induced changes in both tissues. In the liver, it stimulated α-KGDH and GDH activities, whereas in the brain it decreased α-KGDH activity while increasing GDH activity. Therefore, atorvastatin exerted beneficial effects in both tissues.

## 4. Material and Methods

### 4.1. Animals and Experimental Design

Male Wistar rats (total *n* = 24), aged 6–7 weeks and weighing 150–170 g, were selected for this study. The animals were housed in pairs in autoclavable polycarbonate cages, with free access to food and water. The animal room was maintained at a controlled temperature of 22 ± 2 °C and humidity of 50 ± 10%, under a 12 h light/dark cycle (lights on at 07:00 and off at 19:00). All rats underwent a 5-day acclimation period prior to the start of the experiment.

At the beginning of the study, the animals were randomly assigned to two experimental groups. Eight rats (ST group, standard control, *n* = 8) received a standard maintenance laboratory diet (cat. no. 1324, Altromin, Lage, Germany). The remaining rats (CED group, *n* = 16) were fed a cholesterol-enriched diet (standard diet supplemented with 2% cholesterol, Altromin, Lage, Germany) to induce hypercholesterolemia. Group sizes were determined according to published recommendations for animal studies [27]. After eight weeks, blood was collected from the tail vein, and serum total cholesterol (TC) was measured using a colorimetric assay (BioMaxima, Lublin, Poland).

Following confirmation of hypercholesterolemia in the CED group (results published in detail in the paper by Drag et al. [11]), the CED rats were further subdivided into two groups: the HC+A group (*n* = 8), which received atorvastatin at 20 mg/kg/b·wt/day (atorvastatin calcium salt; cat. no. A2476, Tokyo Chemical Industry, Tokyo, Japan), and the HC group (hypercholesterolemic control, *n* = 8), which received the vehicle only (0.15% methylcellulose solution, Merck Life Science, Poznań, Poland). Rats in the ST group also received the vehicle. Drugs or vehicle were administered once daily by oral gavage for three weeks.

The atorvastatin dose of 20 mg/kg b·wt/day was chosen as it has been used in rodent studies investigating the effects of atorvastatin on different enzymes, including α-KGDH in rodent models of hypercholesterolemia [10,28]. The primary objective of this study was to investigate the initial metabolic effects of atorvastatin treatment in brain tissue rather than long-term functional outcomes. Therefore, a 3-week treatment period was chosen to identify early changes in selected mitochondrial dehydrogenases. In addition, this timeframe is supported by previous findings showing that atorvastatin administered at 20 mg/kg b·wt/day for 3 weeks significantly improved mitochondrial respiratory enzyme activities in the brain as well as α-KGDH and GDH in liver [10,29].

### 4.2. Tissue Collection

On the last day of the experiment, the rats were euthanized by decapitation. Blood was collected, and whole brains were excised and immediately freeze-clamped using aluminum tongs pre-cooled in liquid nitrogen and subsequently frozen at −80 °C for analyses. For the determination of a specific parameter an appropriate amount of frozen brain tissue from each rat was sampled and homogenized in relevant buffer (details described below).

### 4.3. Determination of α-KGDH Activity

All chemicals required for the determination of α-KGDH activity were obtained from Merck Life Science (Poznań, Poland). Tissue extracts for the assessment of cerebral α-KGDH activity were prepared as previously described [30]. In brief, frozen whole brain tissue (approximately 100 mg) was pulverized in liquid nitrogen, then weighed and homogenized in an appropriate extraction buffer (containing 30 mM 4-(2-hydroxyethyl)-1-piperazineethanesulfonic acid (HEPES), 3% Triton™ X-100, 2 mM ethylenediaminetetraacetic acid (EDTA), 5 mM dichloroacetic acid (DCA), 50 mM potassium fluoride (KF), 2% bovine serum (BS), 0.01% N-tosyl-L-phenylalanine chloromethyl ketone (TPCK), trypsin inhibitor (10 μg/mL), 1 μM leupeptin hydrochloride, 5 mM dithiothreitol (DTT), 0.5 mM thiamine pyrophosphate (TPP), and potassium hydroxide (KOH)). Subsequently, α-KGDH was concentrated from whole tissue extracts by precipitation with 9% polyethylene glycol. Enzyme activity was evaluated spectrophotometrically by monitoring the rate of NADH formation from NAD^+^ at 340 nm in the presence of a saturating concentration of α-KG, the substrate for α-KGDH, using a UV–VIS Cary 100 spectrophotometer (Varian Medical Systems, Mulgrave, Australia). One unit of α-KGDH activity was defined as the amount of enzyme catalyzing the production of 1 μmol of NADH per minute.

### 4.4. Determination of GDH Activity

In brief, approximately 50 mg of frozen whole brain tissue was grounded in liquid nitrogen, weighed, homogenized on ice in phosphate buffer (pH 8.00) containing 10 mM KH_2_PO_4_, 10 mM K_2_HPO_4_, 0.1 mM, EDTA, and centrifuged (20,000× *g*, 4 °C). The GDH activity (in the direction of reductive amination of α-KG) was determined in supernatants using the spectrophotometric method by measuring the rate (at 340 nm) of NADH generation from NAD^+^ using the saturating concentration of α-KG, a substrate for GDH (UV-VIS Cary 100 spectrophotometer Varian Medical Systems, Mulgrave, Australia) [31]. The GDH activity was expressed as mU/mg protein. Protein concentration was determined using commercial colorimetric assay (Total Protein Kit; Biuret Method; BioMaxima, Lublin, Poland).

### 4.5. Determination of IDH Activity

Briefly, approximately 50 mg of frozen whole brain tissue was grounded in liquid nitrogen, weighed, homogenized on ice in phosphate buffer (pH 8.00) containing 10 mM KH_2_PO_4_, 10 mM K_2_HPO_4_, 0.1 mM EDTA and centrifuged (20,000× *g*, 4 °C). The IDH activity was determined according to Bubber et al. [32] in supernatants using the spectrophotometric method by measuring the rate (at 340 nm) of NADPH generation from NADP+ using the saturating concentration of isocitrate as a substrate for IDH (UV-VIS Cary 100 spectrophotometer Varian Medical Systems, Mulgrave, Australia). The IDH activity was expressed as mU/mg protein. Protein concentration was determined using commercial colorimetric assay (Total Protein Kit; Biuret Method; BioMaxima, Lublin, Poland).

### 4.6. Determination of Glutamate Level

Briefly, approximately 30 mg of frozen whole brain tissue was grounded in liquid nitrogen and homogenized in 10 volumes (*w*/*v*) of phosphate-buffered saline (PBS). The homogenates were centrifuged (20,000× *g*, 4 °C), and the supernatants were collected for further analysis. An aliquot of 20 μL of each supernatant was used for glutamate determination according to the manufacturer’s protocol. Glutamate concentration was determined using a commercial colorimetric enzymatic assay kit (MAK330, Merck Life Science, Poland) based on GDH-catalyzed oxidation of glutamate. The intensity of the generated color, measured spectrophotometrically at 565 nm (BioTek Reader EL 808, BioTek Instruments, Winooski, VT, USA) was proportional to the glutamate concentration in the sample.

### 4.7. Western Blot Analysis

Relative protein expression of α-KGDH E1 and E2 subunits, GDH, and IDH were determined by Western blot. Frozen brain tissue samples were grounded into a fine powder with a nitrogen-cooled mortar and pestle and homogenized using manual homogenizers in ice-cold buffer containing 20 mM Tris (pH 7.8), 137 mM NaCl, 2.7 mM KCl, 1 mM MgCl_2_, 1% Triton X-100, 10% (*w*/*v*) glycerol, 1 mM EDTA, 1 mM dithiothreitol, supplemented with protease and phosphatase inhibitor cocktail (Thermo Fisher Scientific, Waltham, MA, USA). Homogenates were incubated on ice for 1 h under gentle agitation and then centrifuged for 15 min at 15,000 rpm 4 °C. The supernatants were transferred into fresh tubes. The protein concentration was measured using UV-VIS Cary 100 spectrophotometer (Varian Medical Systems, Mulgrave, Australia) and Total Protein Kit (BioMaxima, Lublin, Poland). Equal amounts of protein (30 μg total protein) were loaded into 12% SDS polyacrylamide gels and separated. After electrophoresis, proteins were transferred into nitrocellulose membranes (Amersham Hybond™, GE™ Healthcare, Pittsburgh, PA, USA), at a constant voltage (25 V) in transfer buffer (25 mM Tris, 192 mM glycine, pH 8.3, with 20% methanol). Blots were washed with TBST (150 mM NaCl, 50 mM Tris, 0.05% Tween 20), blocked with 5% skimmed milk in TBST, and incubated overnight at 4 °C with the appropriate primary antibodies against E1 (OGDH cat. #PA5-88571, Invitrogen, Thermo Fisher Scientific, Waltham, MA, USA), E2 (DLST, cat. CSB-PA861526LA01HU, Cusabio Technology LLC, Houston, TX, USA), GDH (Glud1 cat. #PA5-121121, Invitrogen, Thermo Fisher Scientific, Waltham, MA, USA) and IDH (cat. A7190, Abclonal, Woburn, MA, USA). All primary antibodies were used in dilution 1:1000. After incubation with primary antibodies, membranes were washed with TBST and incubated with secondary antibodies, goat anti-rabbit IgG-H&L (HRP) (cat. ab6721, Abcam, Cambridge, UK, used in dilution 1:7000). The protein bands were visualized using an enhanced chemiluminescence method (Clarity Western ECL Substrate BioRad, Hercules, CA, USA) with GeneSys G:Box Chemi-XR5 (Syngene, Cambridge, UK). The values of optical density obtained for E1 and E2 were normalized using Stain-Free total protein normalization (Pierce™ Reversible Protein Stain Kit for Nitrocellulose Membranes, Thermo Fisher Scientific, Waltham, MA, USA) and presented as relative protein levels. Raw immunoblots are provided in the Supplementary Materials.

### 4.8. Statistics

Data are presented as medians with interquartile ranges or means ± SD. Statistical analysis was performed using Graph Pad Prism version 8.0 for Windows, Graph Pad Software (San Diego, CA, USA). Data were analyzed using Student’s *t*-test for unpaired samples. The homogeneity of variance was assessed by Levene’s test. As the data did not meet the assumption of normality, differences between groups were evaluated using the Mann–Whitney U test. To evaluate the dependance between the analyzed parameters, correlation analysis was performed using Pearson’s correlation test. The Pearson correlation coefficient (*r*) was calculated to determine the strength and direction of the linear association between two continuous variables. The analysis was performed separately for each experimental group.

For all obtained results acceptable level of statistical significance was set at *p* < 0.05.

## 5. Conclusions

The present study demonstrated that hypercholesterolemia-induced disturbances were associated with increased cerebral α-KGDH and IDH activities as well as reduced GDH activity and glutamate level in male rats. Changes in α-KGDH and IDH, as key enzymes of the mitochondrial metabolism, may represent an adaptive response of cerebral metabolism aimed at preserving mitochondrial function and preventing functional abnormalities. Short-term treatment with atorvastatin attenuated these metabolic disturbances by suppressing α-KGDH and IDH activities, enhancing GDH activity, and restoring glutamate levels. Taken together, these results demonstrate that atorvastatin mitigates hypercholesterolemia-induced cerebral alterations in mitochondrial dehydrogenases, highlighting a potentially favorable effect of the drug.

### Limitations

The present study has several limitations. Since this is the first study on the effect of atorvastatin on selected dehydrogenases, only one dose of atorvastatin administered for 3 weeks was examined. To fully elucidate the influence of atorvastatin further study employing more doses of the drug and prolonged treatment periods should be undertaken. Extending atorvastatin treatment beyond 3 weeks may provide further insight into its sustained effects on mitochondrial dehydrogenases. Second, the experiments were conducted exclusively in male rats. This approach allowed for us to investigate the early molecular effects of atorvastatin under relatively homogeneous experimental conditions. However, biological sex is known to influence mitochondrial functions [33]. Therefore, the findings of the present study should not be directly extrapolated to females. Future studies including both sexes are necessary to determine whether the observed molecular effects are sex dependent. Another limitation of the study is that enzymes activities and glutamate level were measured in the whole brain tissue rather than in specific brain structures. Considering the regional differences in brain metabolism additional studies are needed to investigate region-specific alterations triggered by atorvastatin.

## Data Availability

The original contributions presented in this study are included in the article/Supplementary Materials. Further inquiries can be directed to the corresponding author.

## Supplementary Materials

The following supporting information can be downloaded at: https://www.mdpi.com/article/10.3390/ijms27156791/s1.
