# Supplementary material for: Atorvastatin Mitigates Hypercholesterolemia-Induced Alterations in Cerebral Mitochondrial Dehydrogenases in Rats
Source: Int J Mol Sci. 2026 Jul 29;27(15):6791. doi: 10.3390/ijms27156791 (PMC13467077; doi:10.3390/ijms27156791)

Supplementary data for:

Atorvastatin mitigates hypercholesterolemia-induced alterations in cerebral mitochondrial dehydrogenases in rats.

Authors

Malgorzata Belczyk, Anna Gawedzka, Jagoda Drag, Oliwia Stelmach, Malgorzata Knapik-Czajka

Affiliation

Department of Biochemical Analytics, Faculty of Pharmacy, Jagiellonian University Medical College, 30-688 Krakow, Poland

Corresponding author: Malgorzata Knapik-Czajka

e-mail address: malgorzata.knapik-czajka@uj.edu.pl

Department of Biochemical Analytics, Faculty of Pharmacy, Jagiellonian University Medical College, 30-688 Krakow, Poland

Supplementary Data.

Original western blot images for protein of interest presented in Fig.2

Prior to immunodetection, images of the untrimmed nitrocellulose membranes showing total protein staining in all lanes (30 µg of brain tissue lysate per lane) were obtained using the Pierce™ Reversible Protein Stain Kit for Nitrocellulose Membranes (Thermo Fisher Scientific). Subsequently, each of the nitrocellulose membranes was trimmed according to the size and position of the bands corresponding to the protein of interest. Next, membranes were incubated with specific antibodies and visualized using an enhanced chemiluminescence method.

The raw, trimmed membranes along with full-size membranes showing total protein staining are presented below. On each membrane image, the marker bands corresponding to the analyzed protein are highlighted. The bands labeled in the red box are presented in the figures in the manuscript. The bands which were not considered in the study, are marked with an "X".

Figure 2A: <u>OGDH (E1 subunit of α-KGDH) - 116 kDa</u>.

ST group:

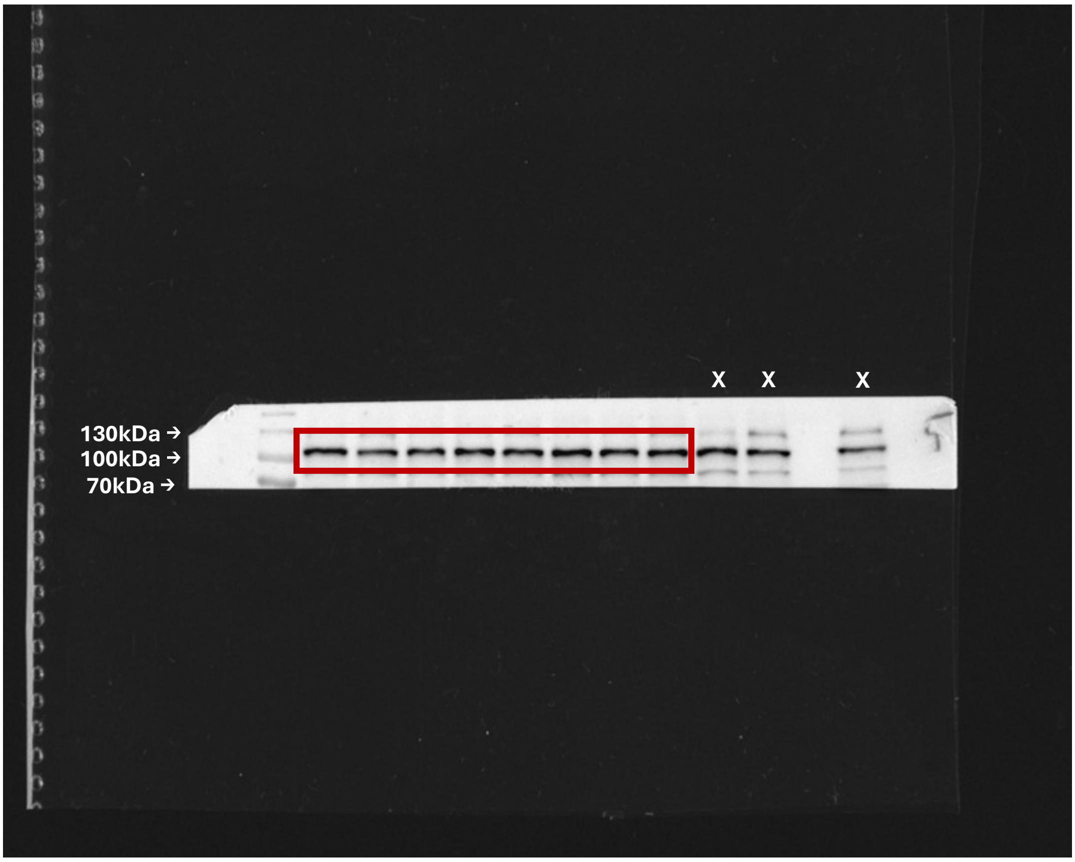

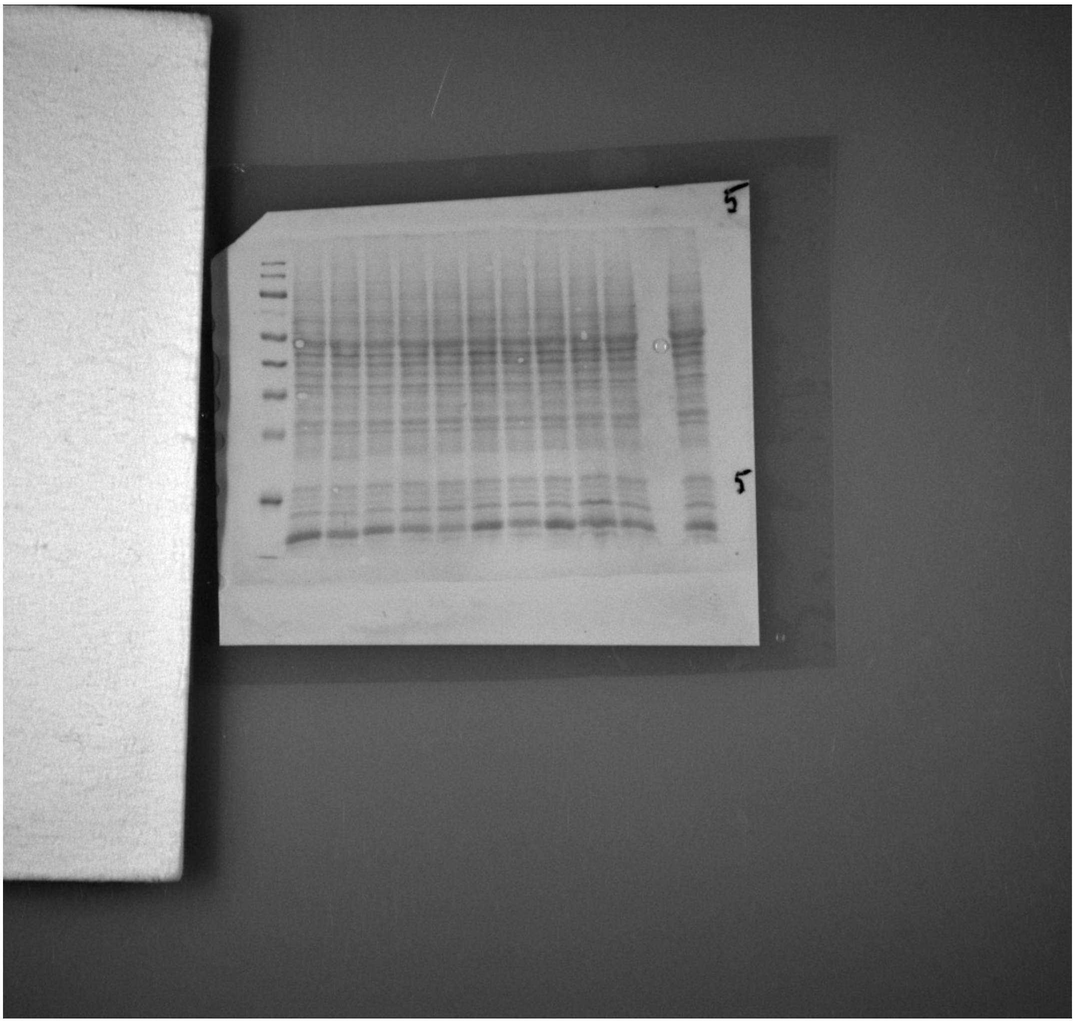

HC group:

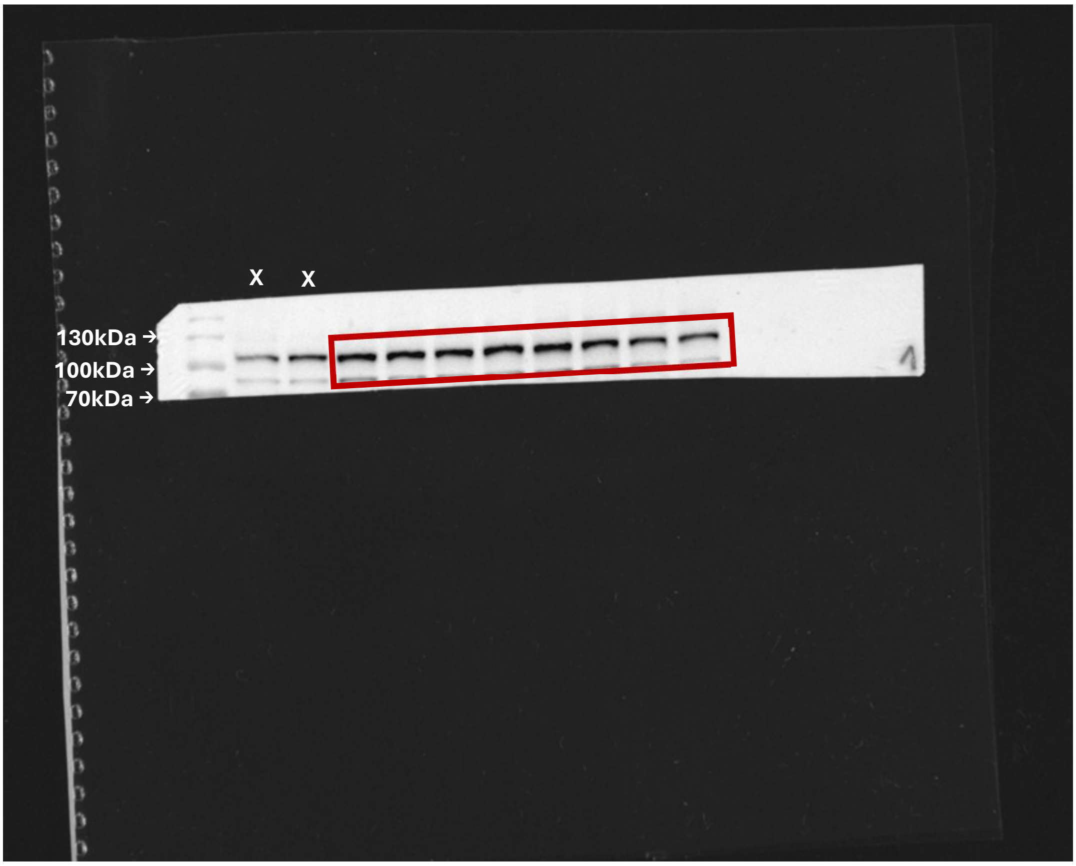

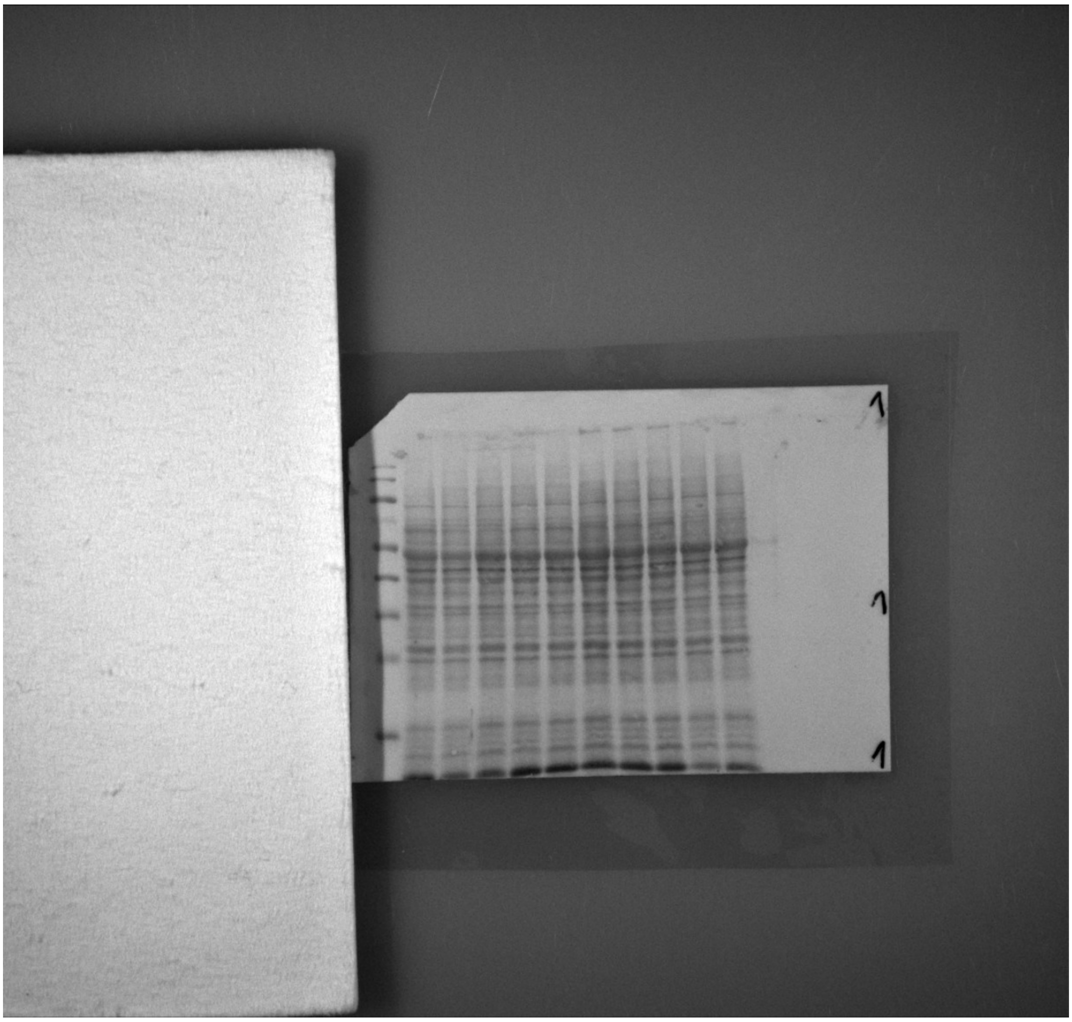

HC+A group:

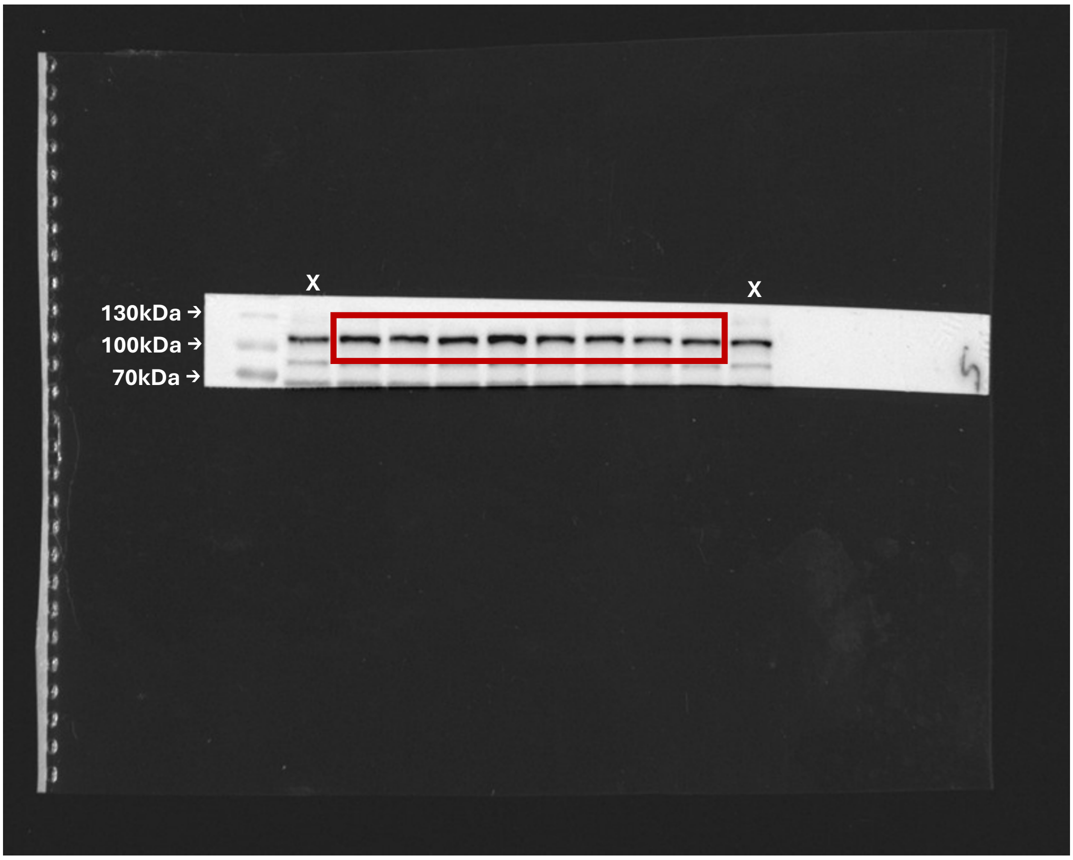

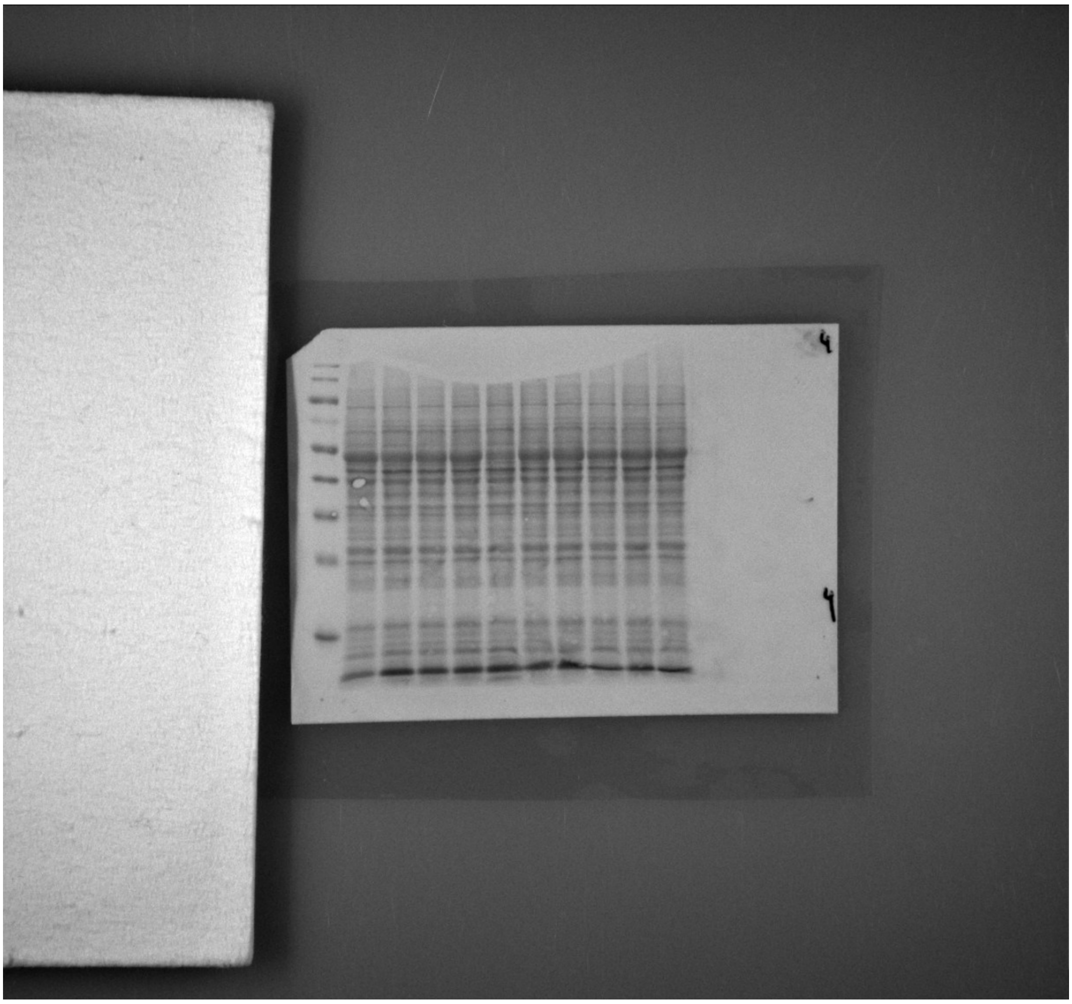

Figure 2B: <u>**DLST (E2 subunit of α-KGDH) - 49 kDa**</u>**.**

ST group:

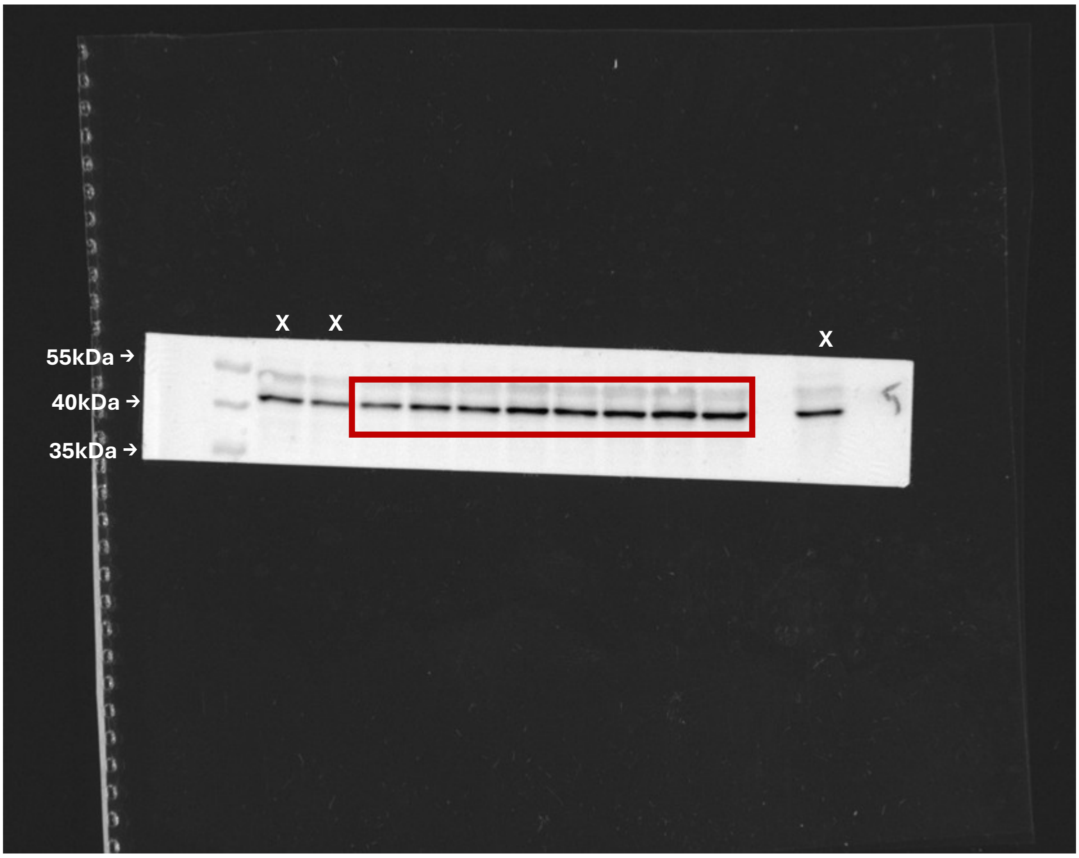

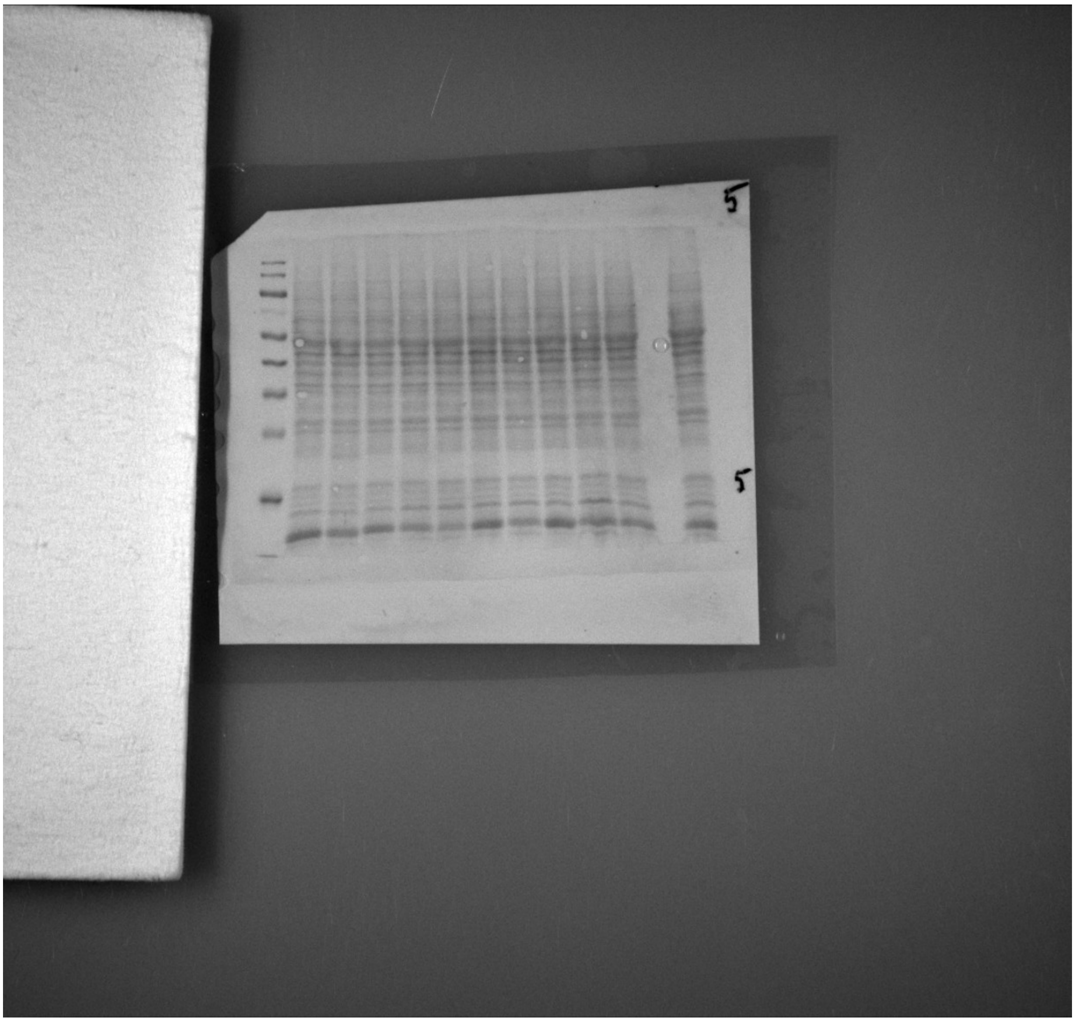

HC group:

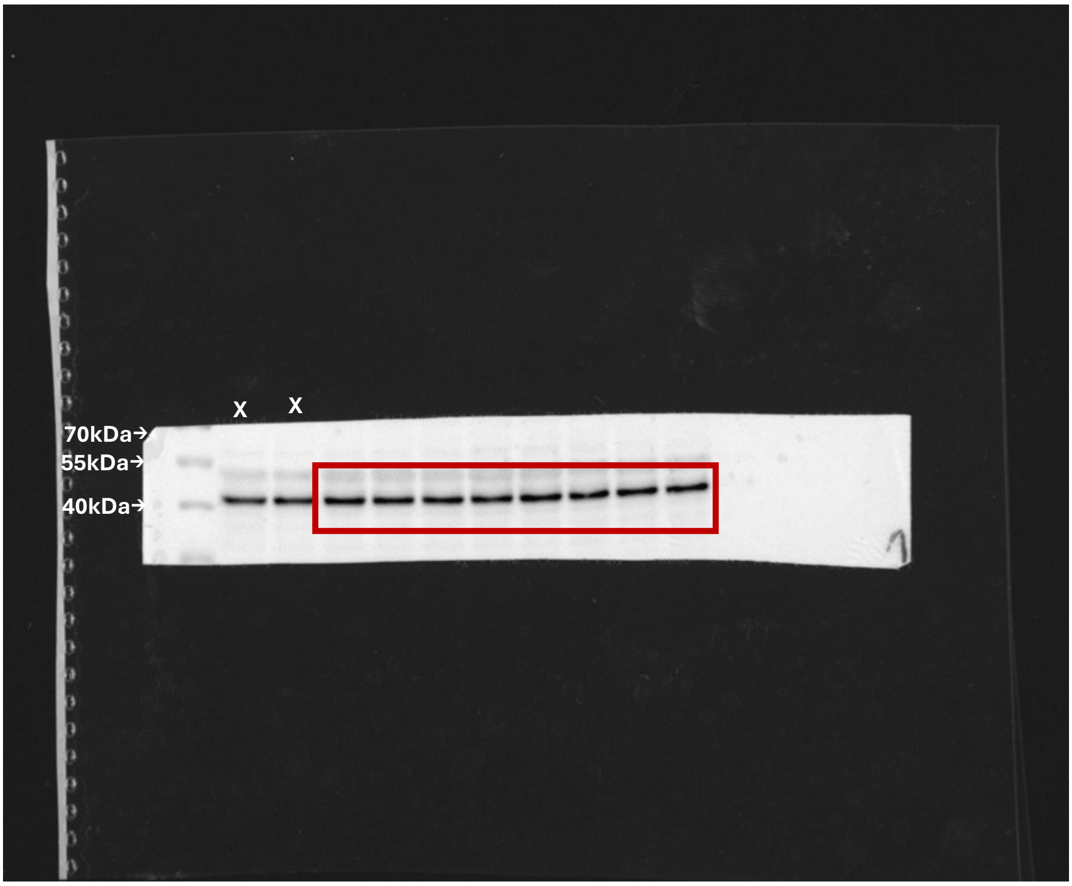

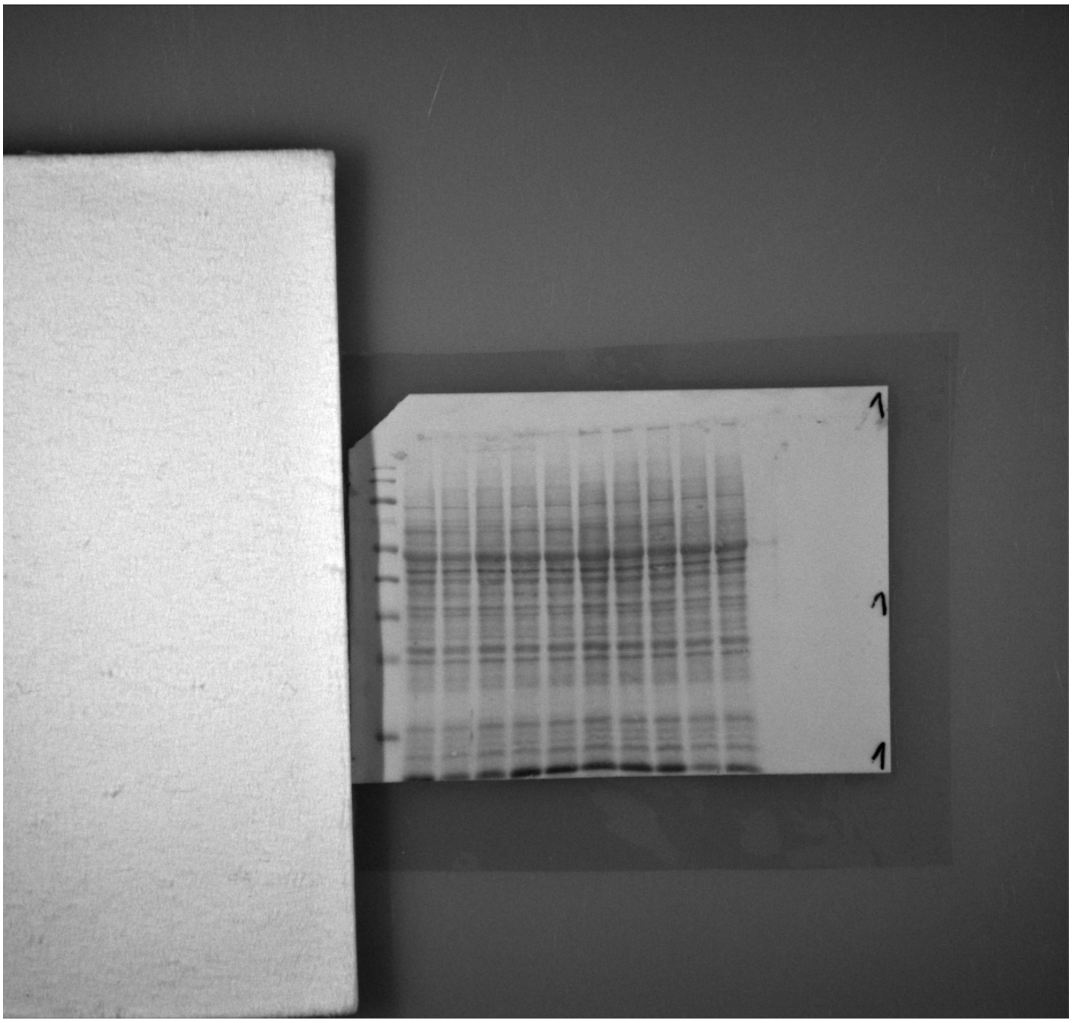

HC+A group:

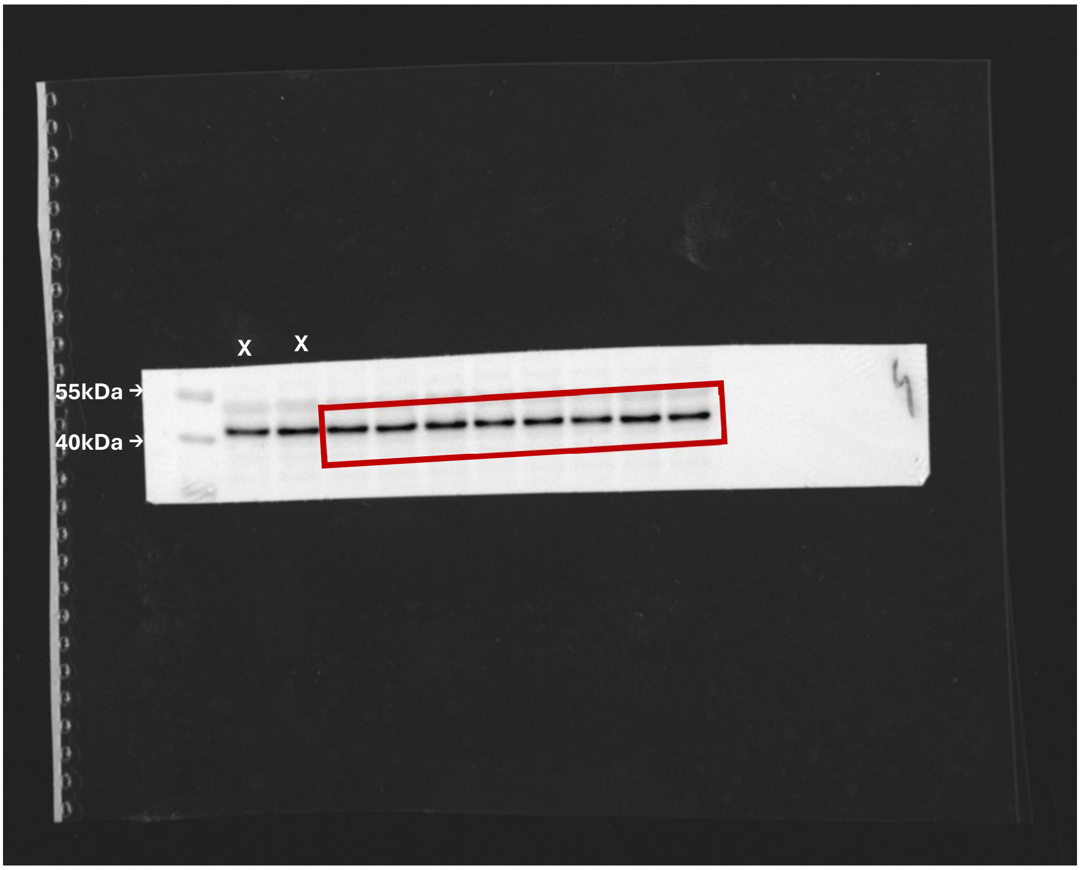

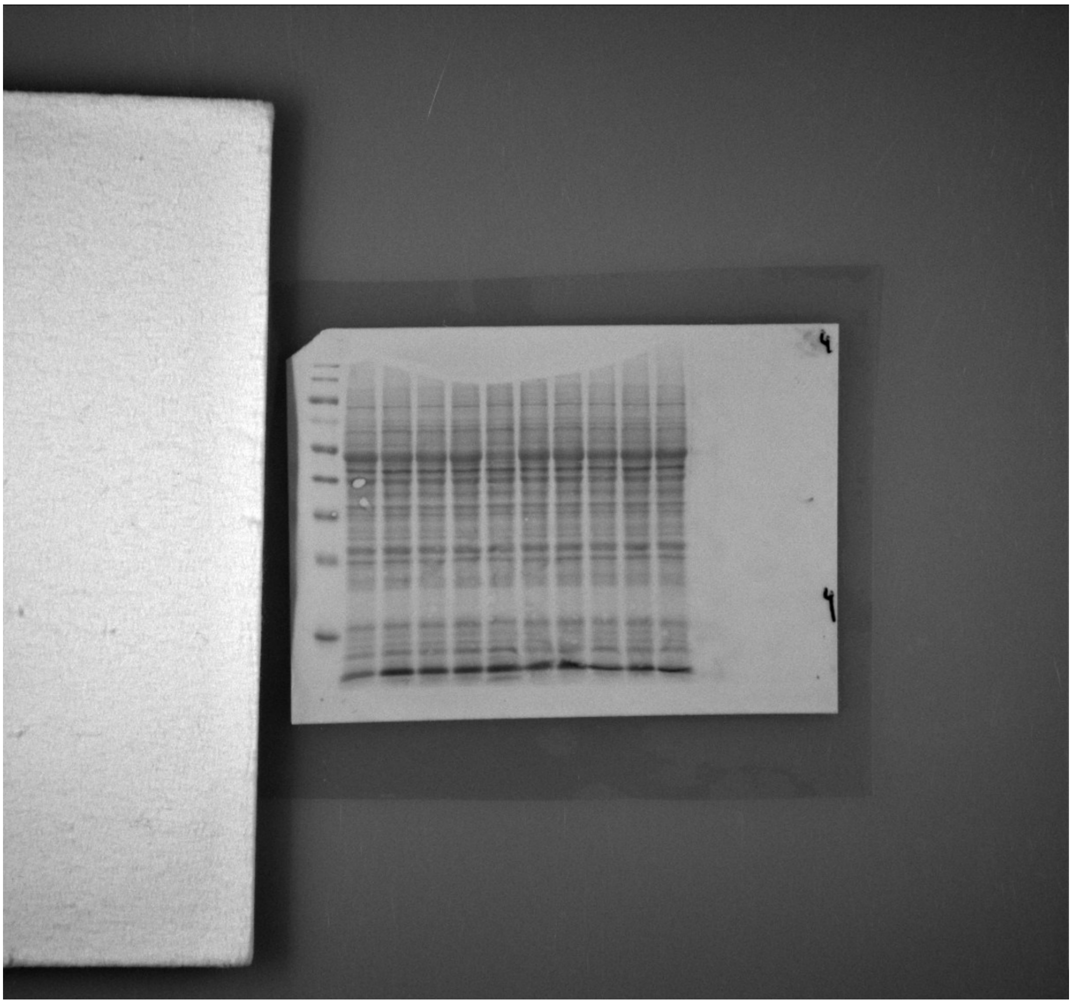

Figure 2C: **<u>IDH – 51kDa</u>.**

ST group:

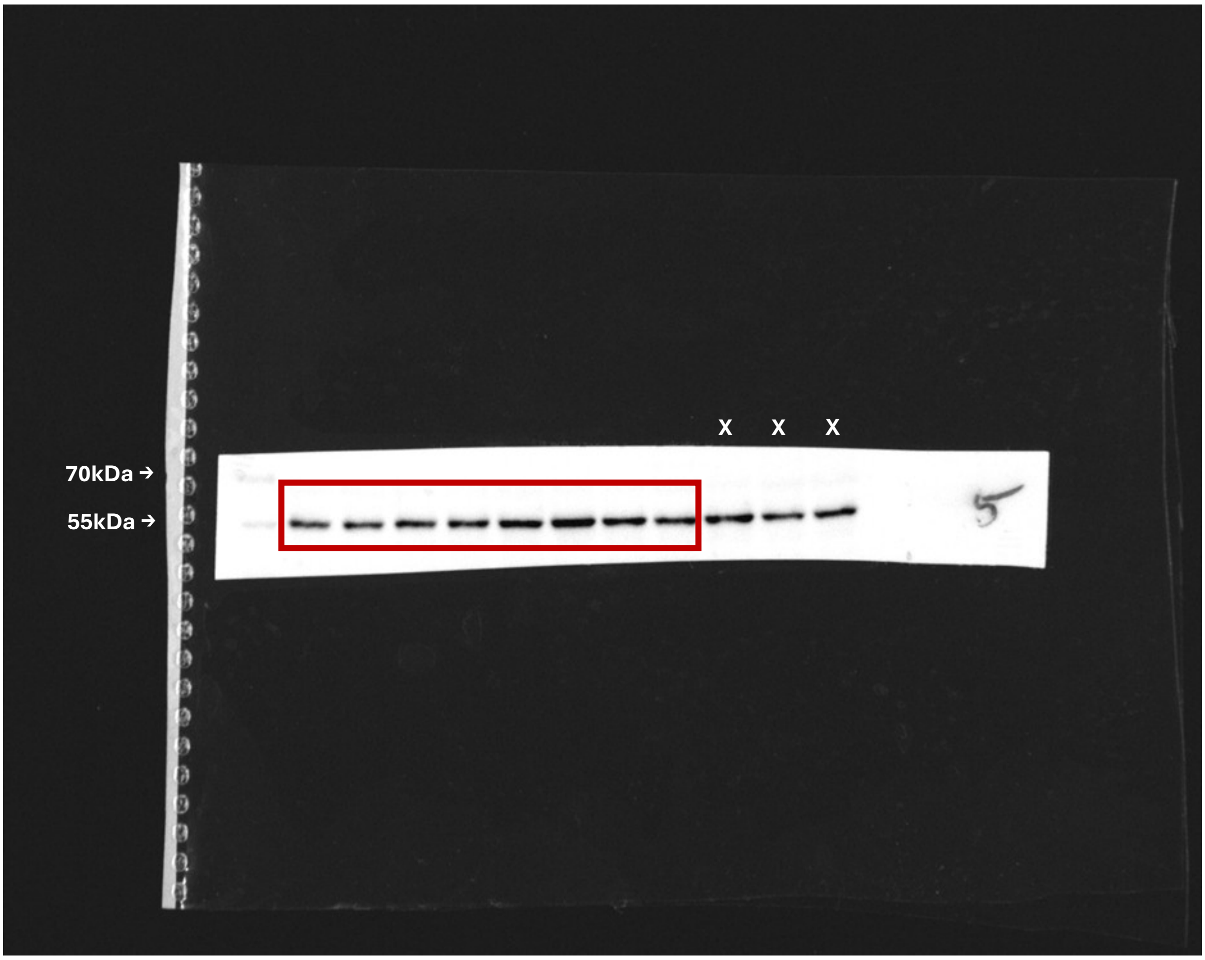

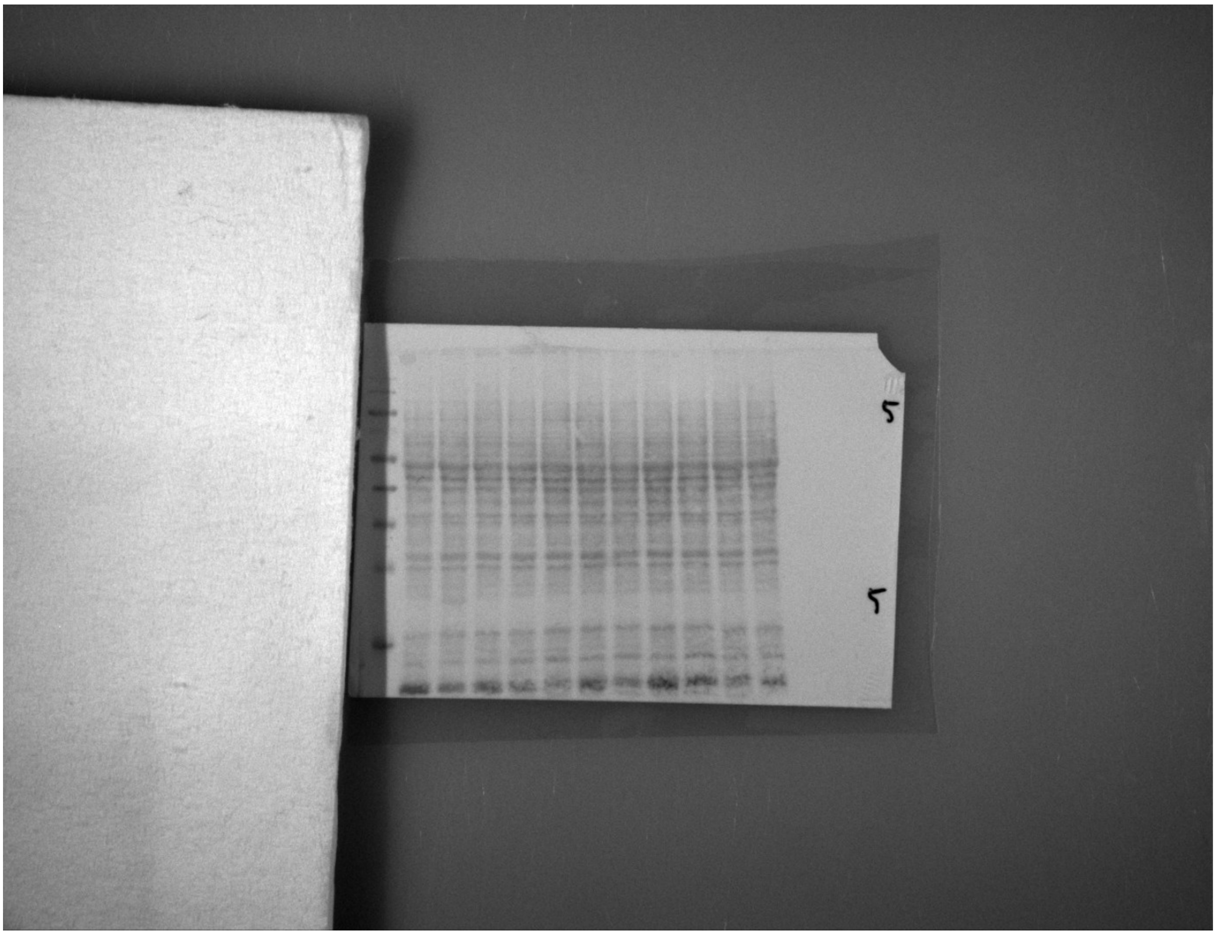

HC group:

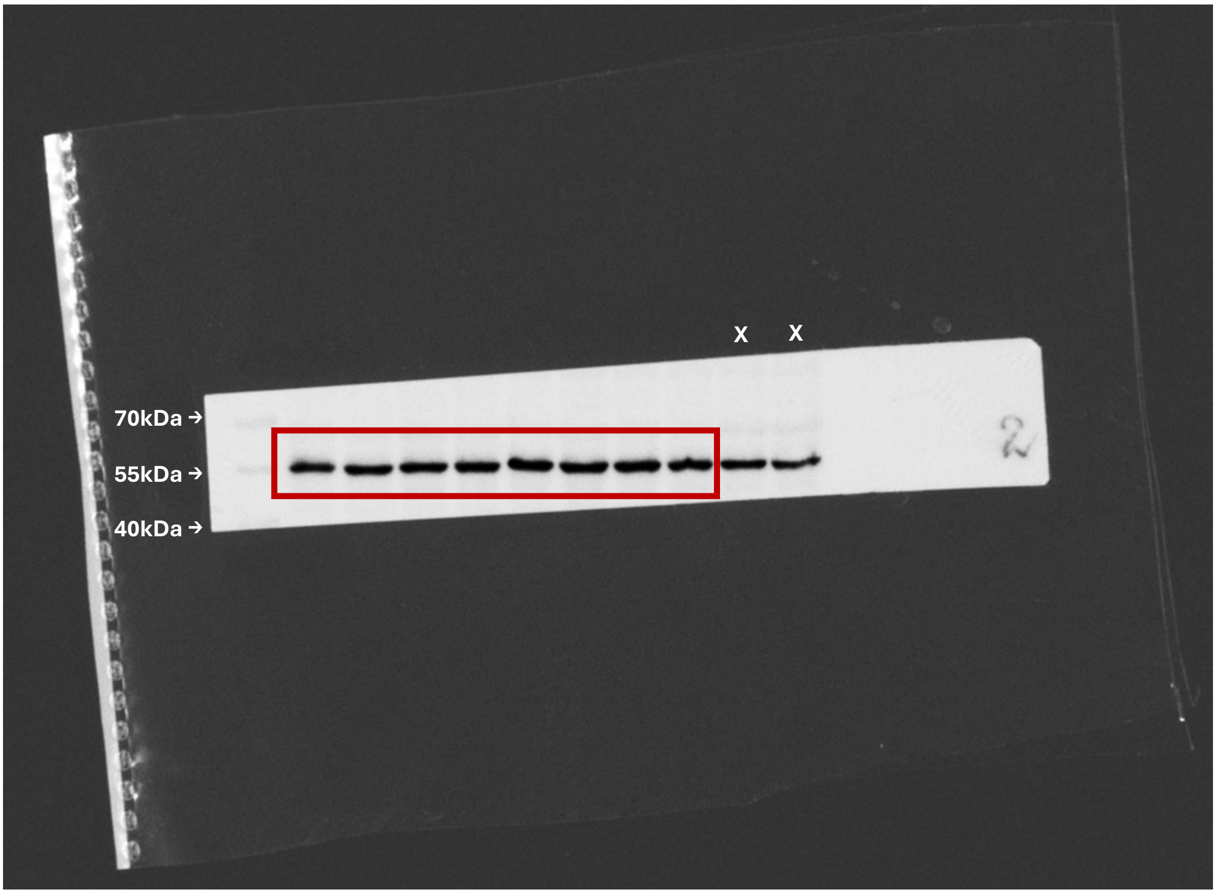

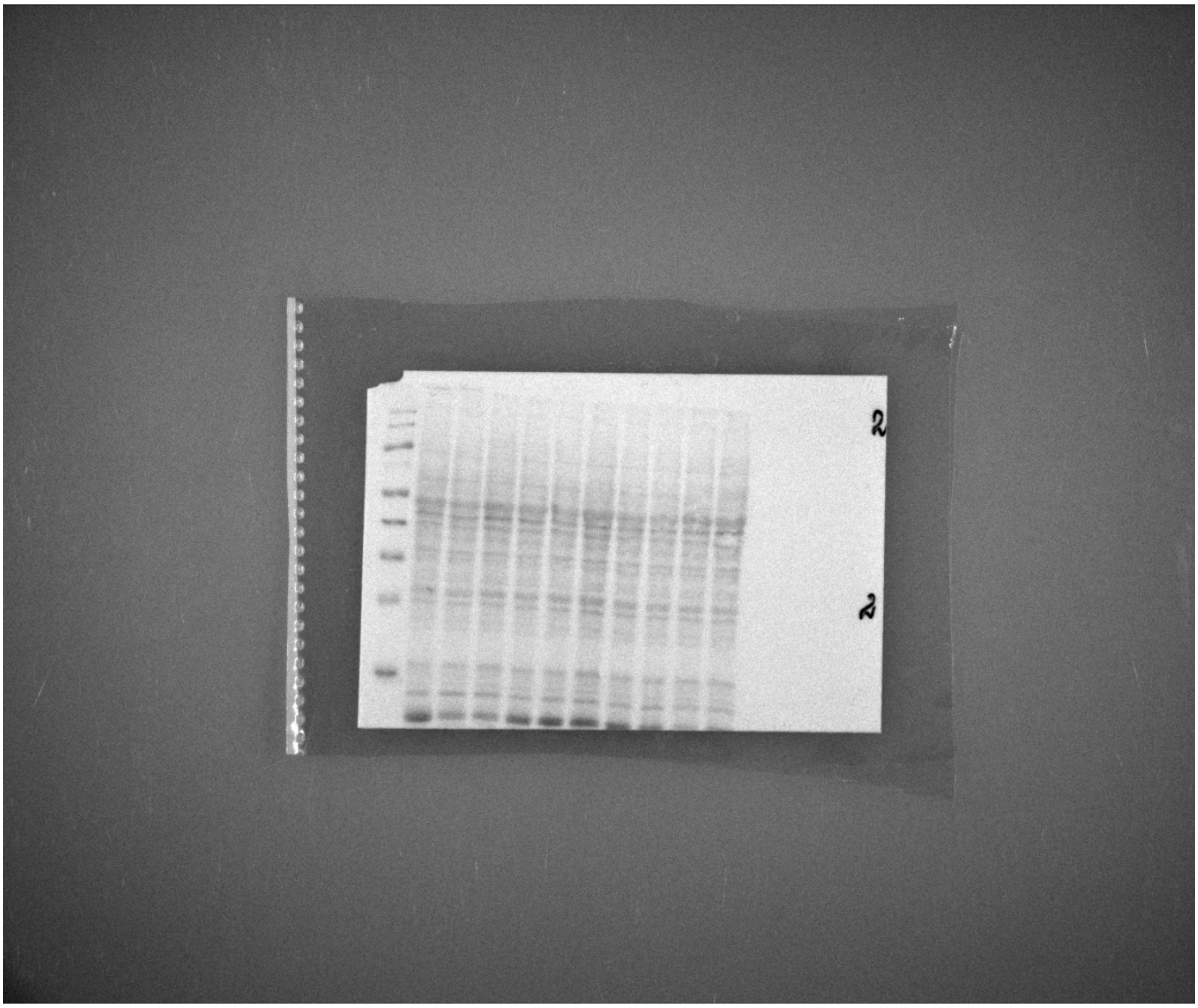

HC+A group:

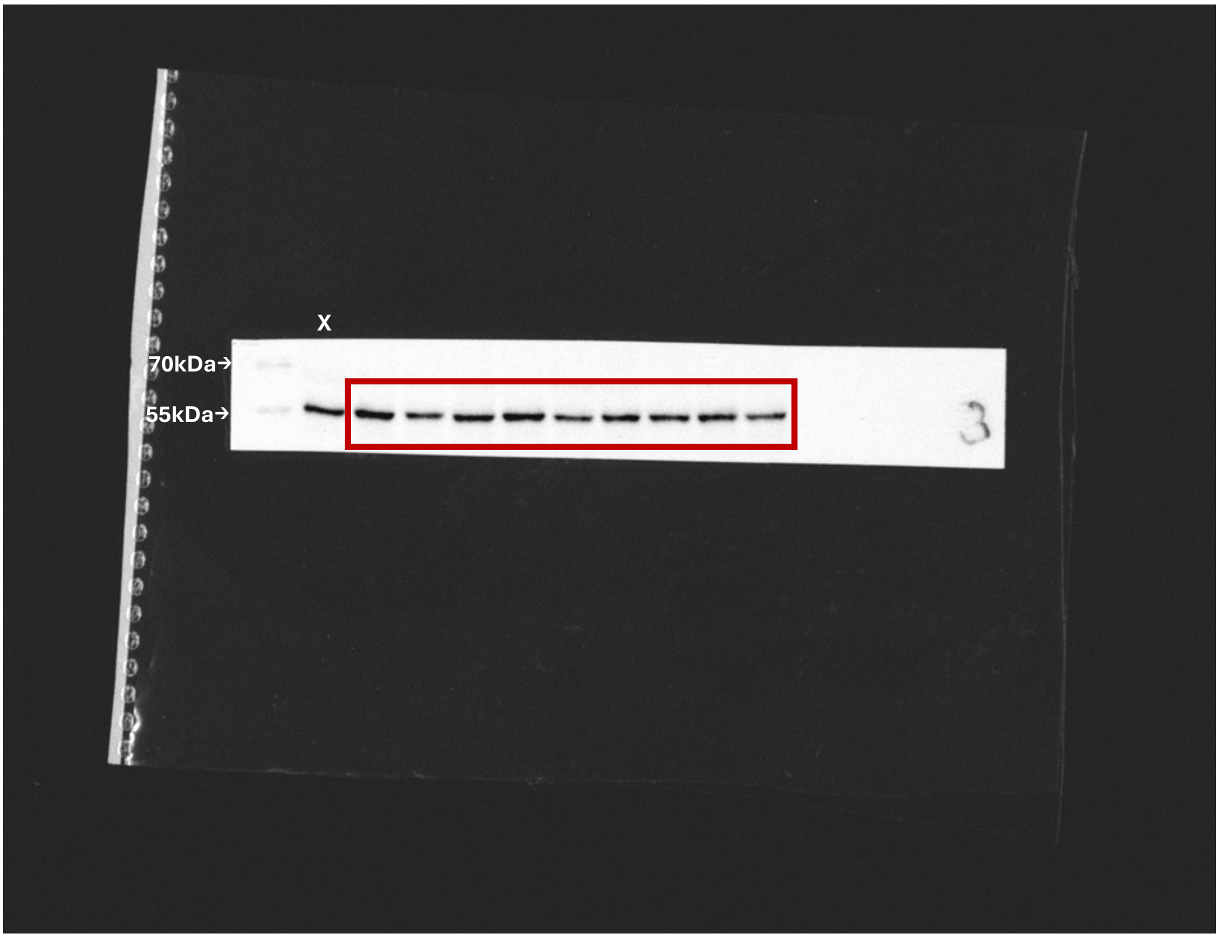

—

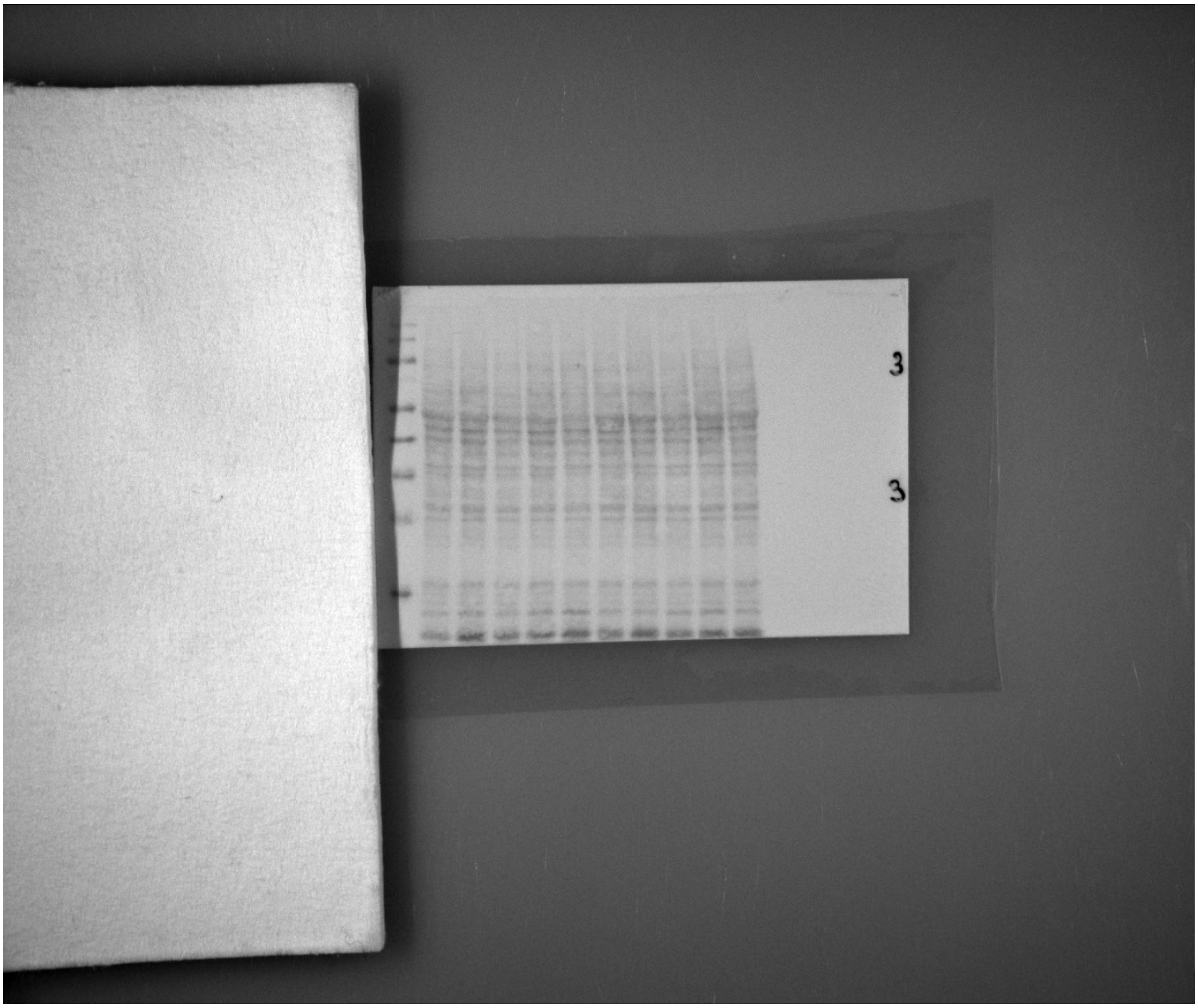

Figure 2D: **Glud1 (GDH) - 50 kDa.**

ST group:

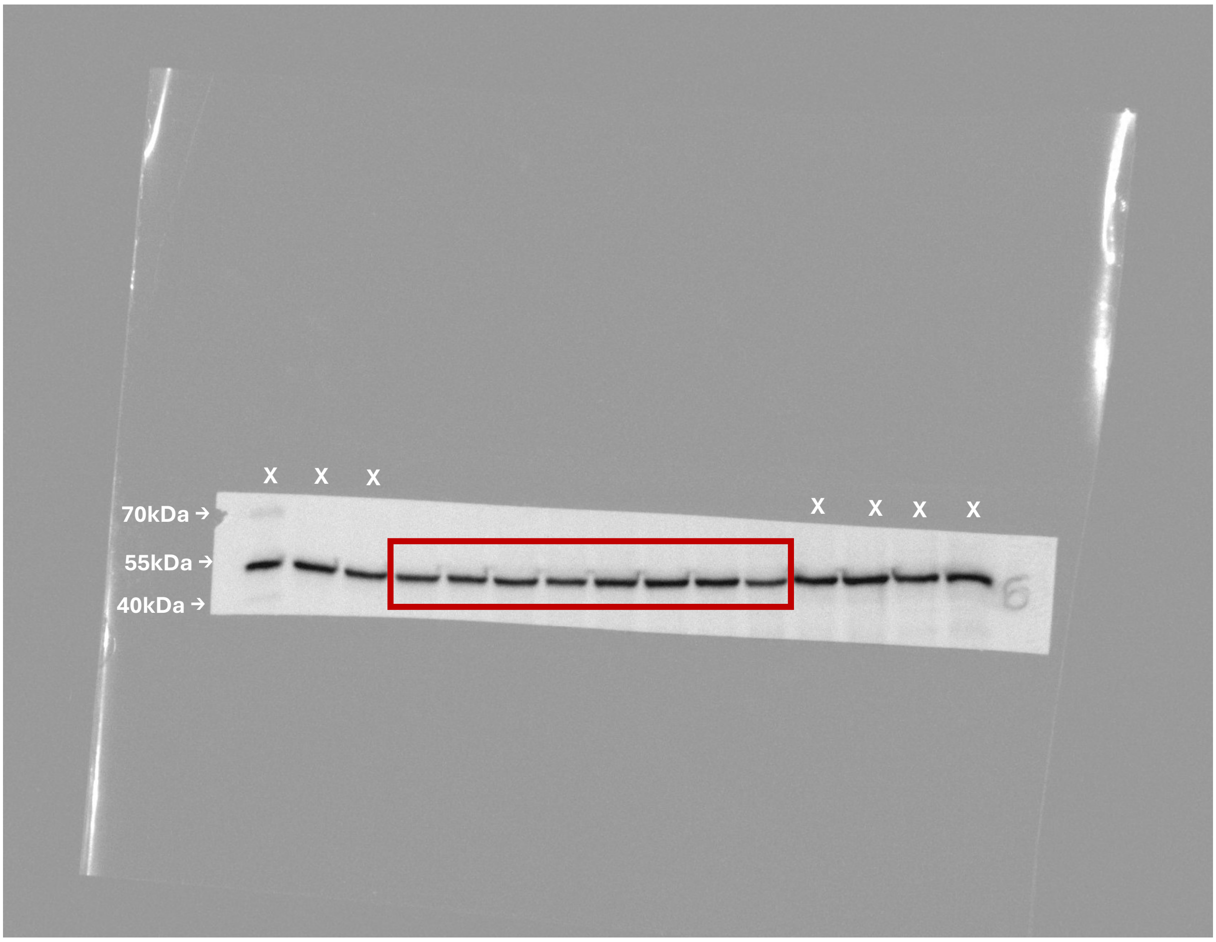

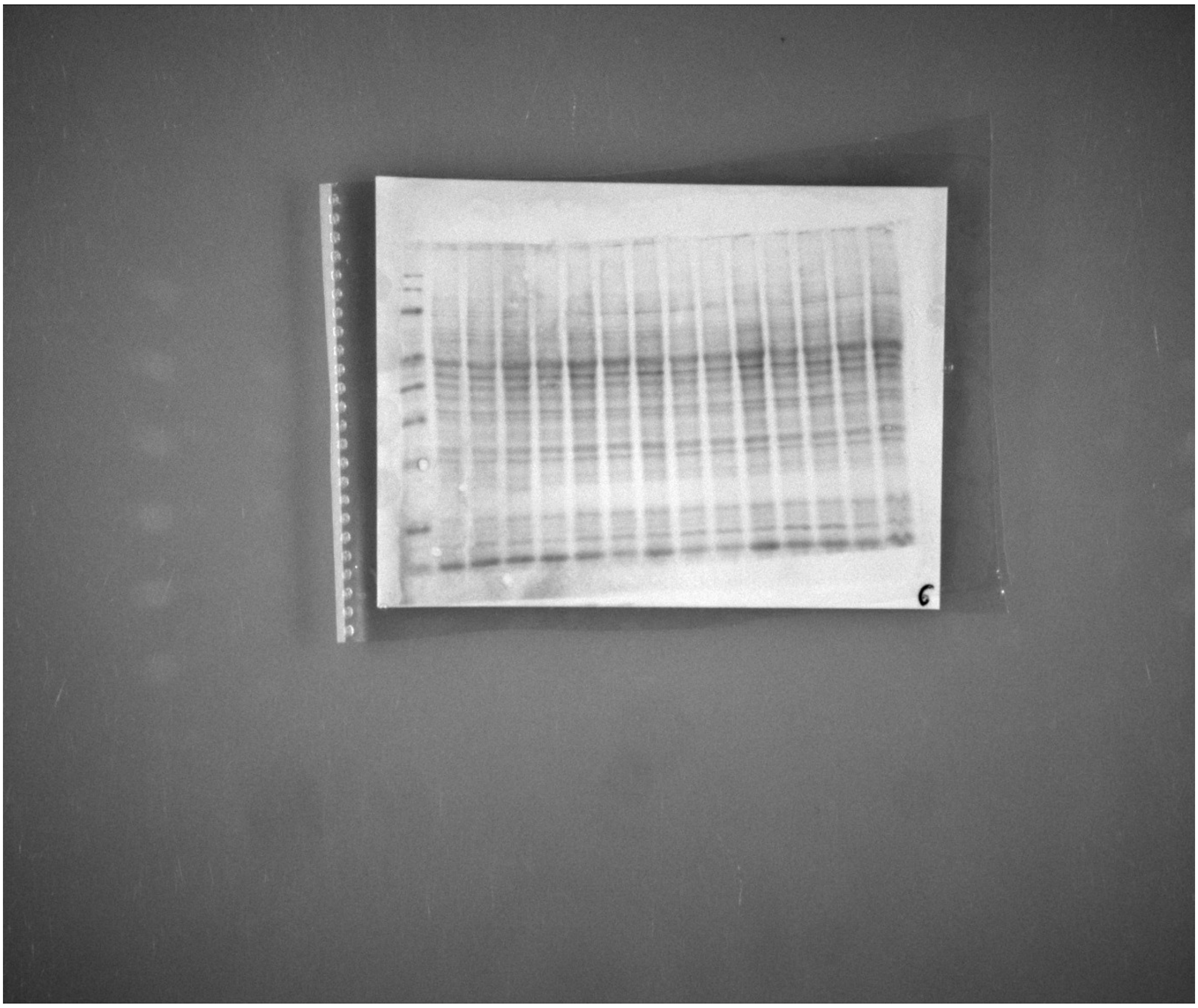

HC group:

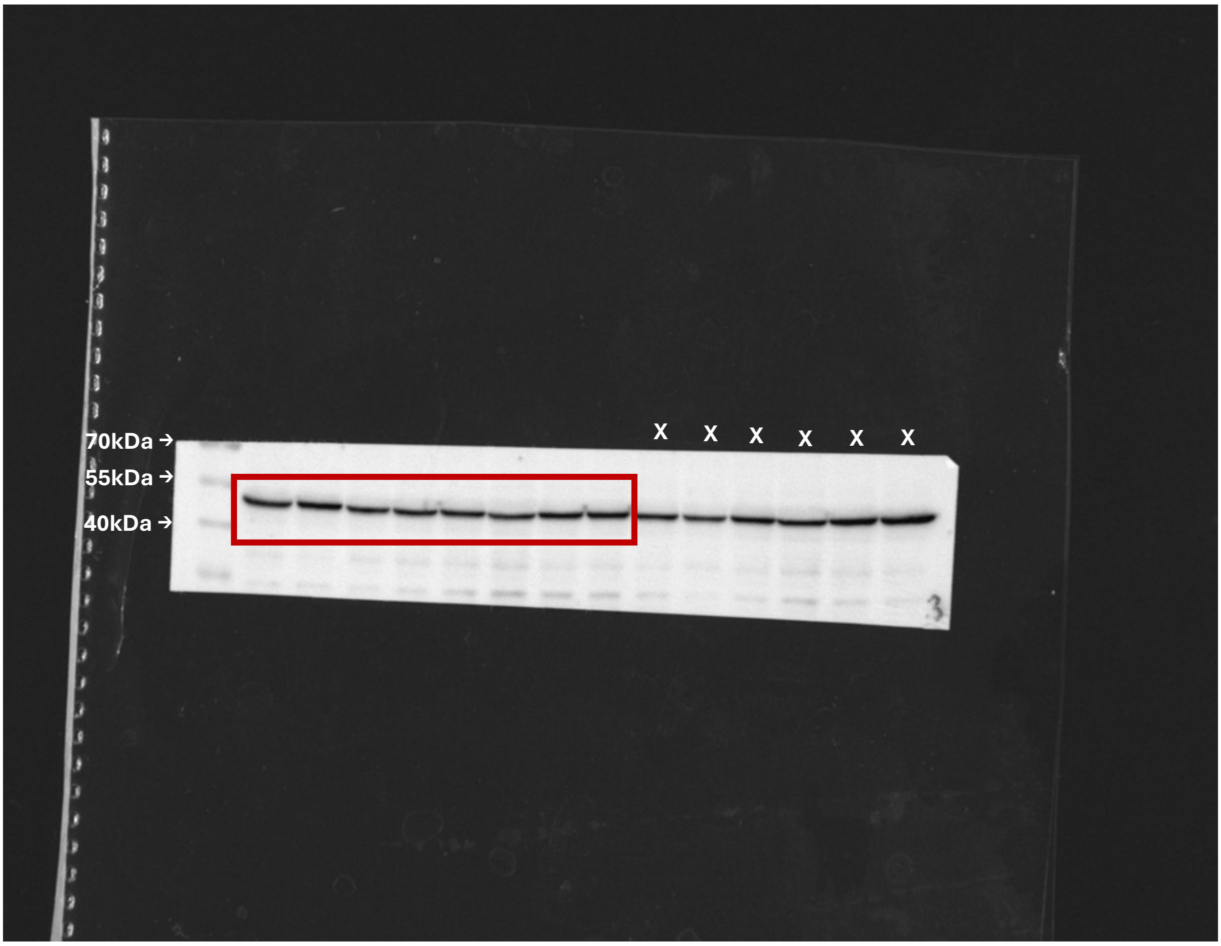

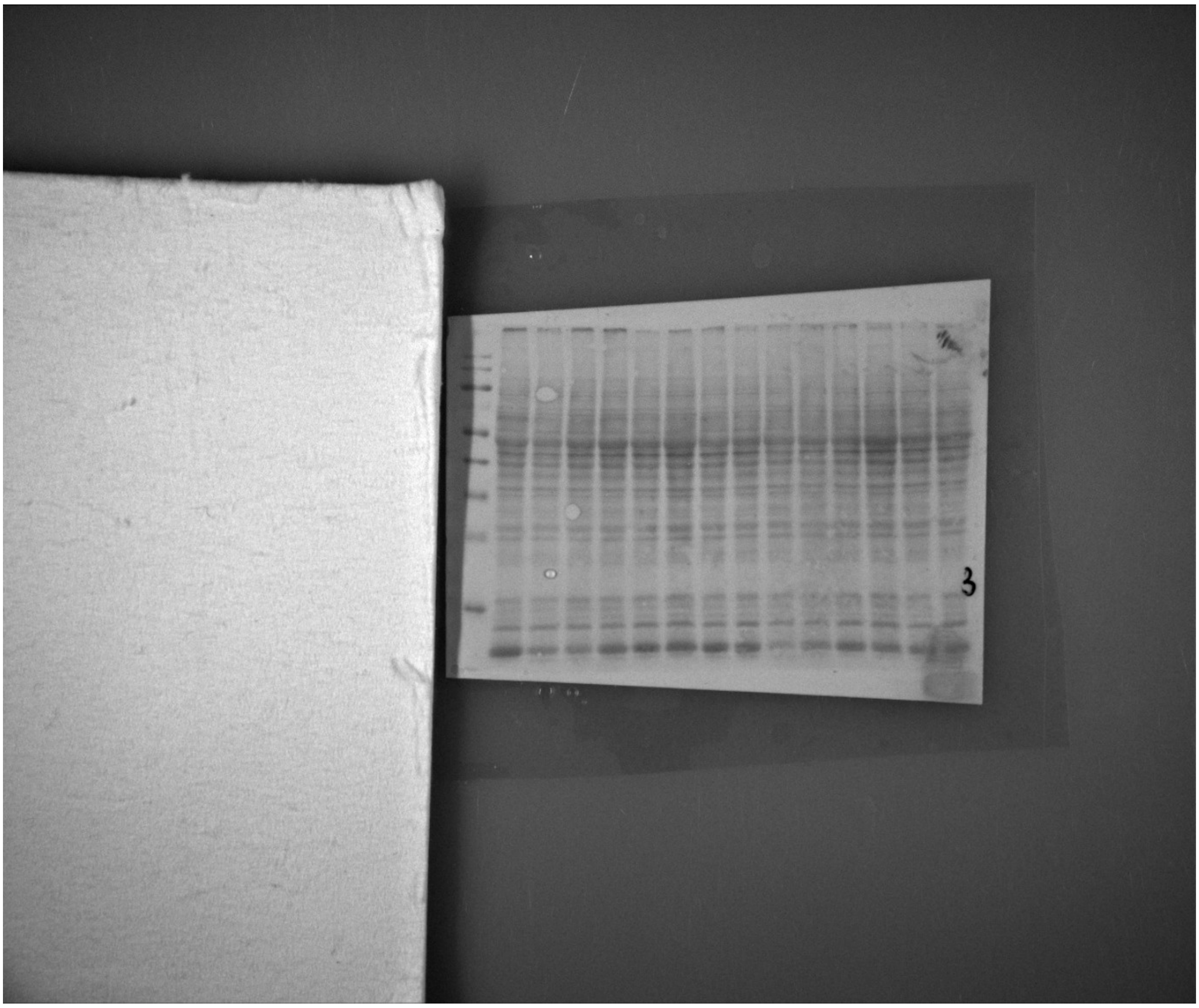

HC+A group:

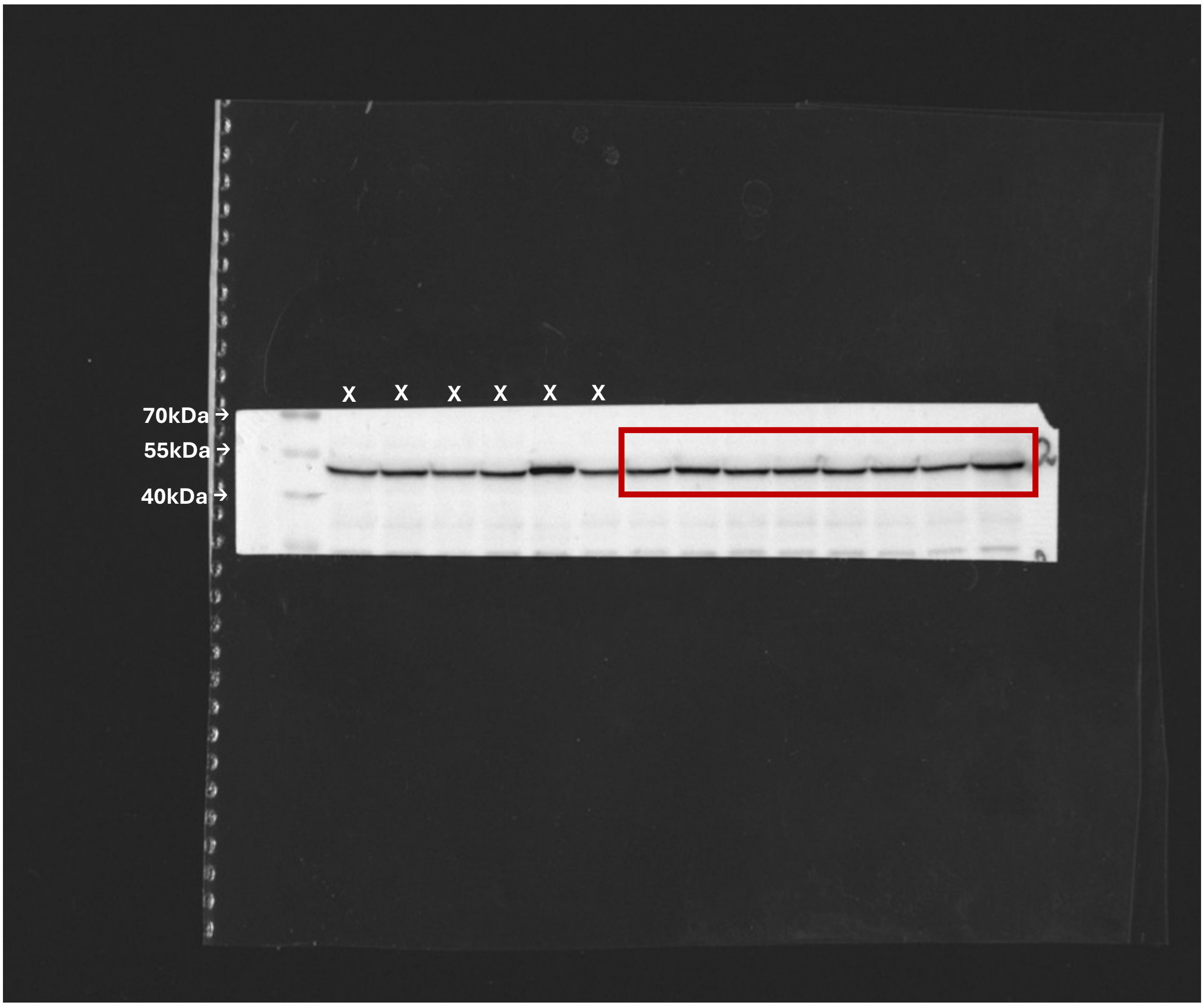

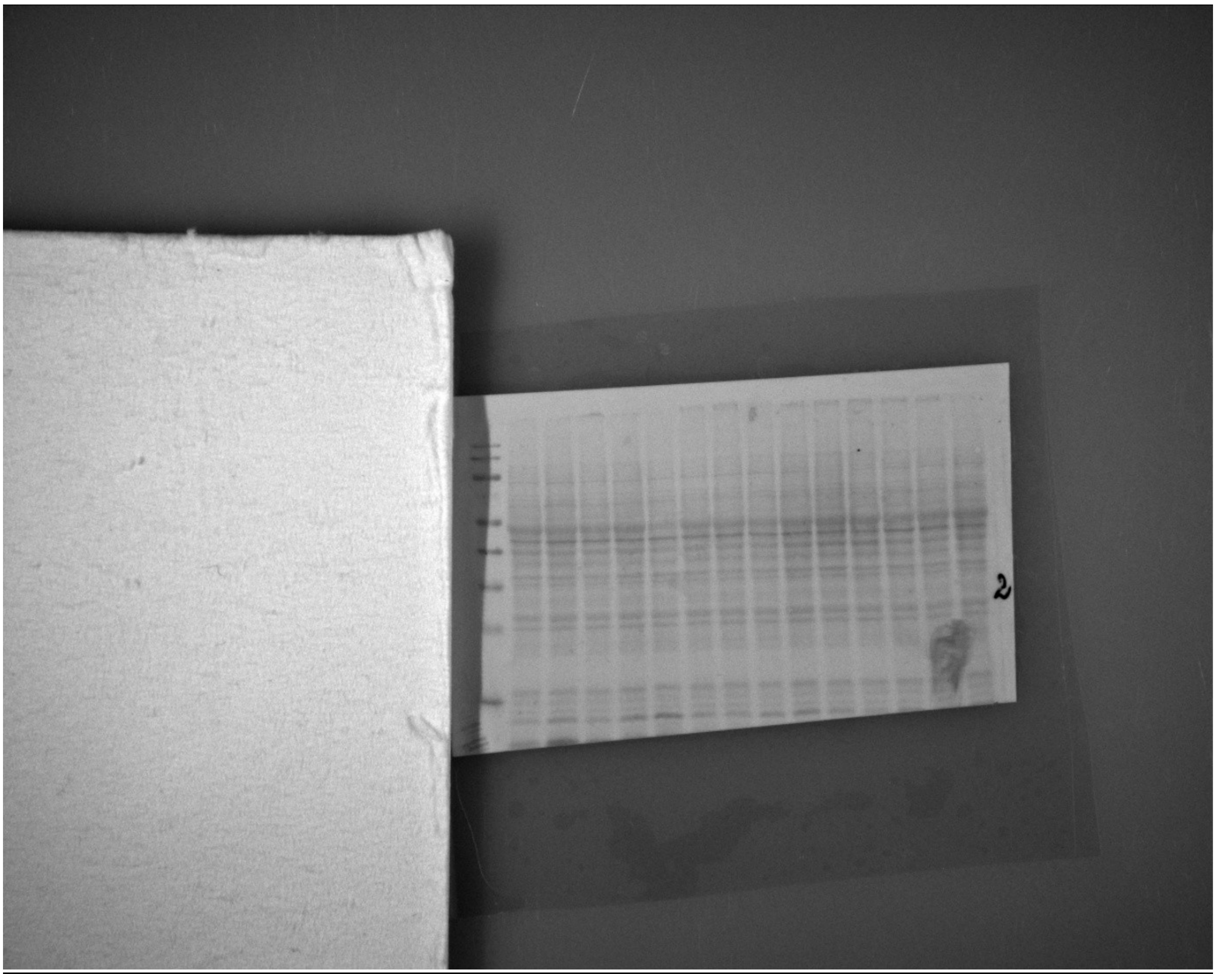

Supplement: Supplementary file 1 [file ijms-27-06791-s001.zip › ijms-4404410-supplementary.pdf]
